# Modeling and optimizing deep brain stimulation to enhance gait in Parkinson’s disease: personalized treatment with neurophysiological insights

**DOI:** 10.1038/s41531-025-00990-5

**Published:** 2025-06-18

**Authors:** Hamid Fekri Azgomi, Kenneth H. Louie, Jessica E. Bath, Kara N. Presbrey, Jannine P. Balakid, Jacob H. Marks, Thomas A. Wozny, Nicholas B. Galifianakis, Marta San Luciano, Simon Little, Philip A. Starr, Doris D. Wang

**Affiliations:** 1 Department of Neurological Surgery, University of California San Francisco, San Francisco, CA USA; 2 Department of Physical Therapy and Rehabilitation Science, University of California San Francisco, San Francisco, CA USA; 3 Department of Neurology, University of California San Francisco, San Francisco, CA USA

**Keywords:** Predictive markers, Parkinson's disease, Basal ganglia, Parkinson's disease, Translational research, Neurophysiology

## Abstract

The effects of deep brain stimulation (DBS) on gait in Parkinson’s disease (PD) are variable due to challenges in gait assessment and limited understanding of stimulation parameters’ impacts on neural activity. We developed a data-driven approach to identify optimal DBS parameters to improve gait and uncover neurophysiological signatures of gait enhancement. Field potentials from the globus pallidus (GP) and motor cortex were recorded in three patients with PD (PwP) using implanted bidirectional neural stimulators during overground walking. We developed a Walking Performance Index (WPI) to assess gait metrics. DBS parameters were systematically varied to study their impacts on gait and neural dynamics. We were able to predict and identify personalized DBS settings that improved the WPI using a Gaussian Process Regressor. Improved walking correlated with reduced pallidal beta power during key gait phases. These findings, along with identified person-specific neural spectral biomarkers, underscore the importance of personalized, data-driven interventions for gait enhancement in PwP. ClinicalTrials.gov registration: NCT-03582891.

## Introduction

Gait disturbances are common symptoms of Parkinson’s disease (PD) and can often manifest as decreased step length^1,2^, increased variability in step length and step time^3–5^, and asymmetry between the two stepping legs^6^. Gait dysfunction reduces mobility, increases fall risk, and significantly impacts a patient’s quality of life^7–10^. While high-frequency deep brain stimulation (DBS) of the basal ganglia is highly effective at mitigating symptoms such as tremors, rigidity, and bradykinesia^11–16^, its impact on gait is more variable and less predictable, with some reports showing improved gait^17–19^, while others indicate no significant improvement or even worsening of gait^20–24^. These unpredictable effects of DBS settings, coupled with individual variability among patients, highlight the need for improved DBS treatments targeting advanced gait-related problems and understanding their impacts on the basal ganglia thalamocortical network neurophysiology^25^.

One significant challenge in enhancing DBS outcomes for treating advanced gait disorders is the lack of a standardized gait metric for clinicians to use during programming. Stride velocity, variability in step time and step length, and arm swing amplitude are among essential gait symptoms seen in patients with PD (PwP)^26–32^. However, focusing on a single metric may overshadow the comprehensive impact of DBS settings, leading to suboptimal outcomes. Currently, DBS programming is mainly conducted with the patient seated, focusing on the assessment of limb motor functions using the Unified Parkinson’s Disease Rating Scale (UPDRS). While some clinicians incorporate walking tests, these are often limited, non-standardized, and based primarily on subjective observation. Moreover, the UPDRS fails to capture many critical aspects of gait dysfunctions specific to PwP, further complicating the evaluation and optimization of DBS settings for gait^33^. Thus, a systematic approach to assessing gait quality is necessary.

Even in cases where gait metrics are evaluated during DBS adjustments, identification of DBS stimulation parameters optimized for gait is difficult^17^. This is because of the extensive parameter space (i.e., amplitude, frequency, and pulse width of the stimulation impulses at each contact); identifying optimal gait settings would place an impractical demand on both time and resources for patients and clinicians^34,35^. Therefore, most clinicians primarily rely on empiric high-frequency stimulation during DBS programming visits for clinical optimization. While high-frequency DBS is usually effective for tremors, rigidity, and bradykinesia, its efficacy for advanced gait disorders is less consistent^36–38^. Lower frequency stimulation can improve gait kinematics, but significant uncertainties remain due to individual variations and inconsistent DBS effects^19,22,24,39–44^. The variability in patient responses to different DBS stimulation parameters underscores the need for a more structured approach to systematically explore the full parameter space and identify settings that optimize gait functions^45–50^.

The next challenge is the limited understanding of gait neurophysiology, which restricts our ability to fully understand the effects of DBS settings on modulating gait-related neural dynamics. Advances in sensing technologies now allow us to explore how changes in DBS settings influence the neural mechanisms driving motor functions^51–54^. By identifying neural biomarkers associated with gait improvements, we would gain (1) valuable insights into the circuits and structures involved in gait control, (2) understanding of how different stimulation parameters can lead to similar improvements in gait, and (3) potentially leverage these biomarkers to guide DBS programming to target specific gait-related oscillations more efficiently to further enhance walking performance. Our previous study has shown that in PwP without gait deficits, low-frequency local field potentials (LFPs) in the subthalamic nucleus and their synchrony with the primary motor cortex change cyclically based on the specific phase of the gait cycle. These dynamic oscillatory changes across the basal ganglia-cortical network may represent a physiologic signature of effective overground walking^55^. Despite these findings, the effects of DBS settings on neural signatures involved in gait are not well studied and may account for the variable effects of DBS on gait. Therefore, a deeper understanding of the relationship between DBS settings, underlying neural mechanisms, and resulting gait outcomes would further enhance our knowledge of the neurophysiological foundations of complex gait functions in PwP.

**Fig. 1:**
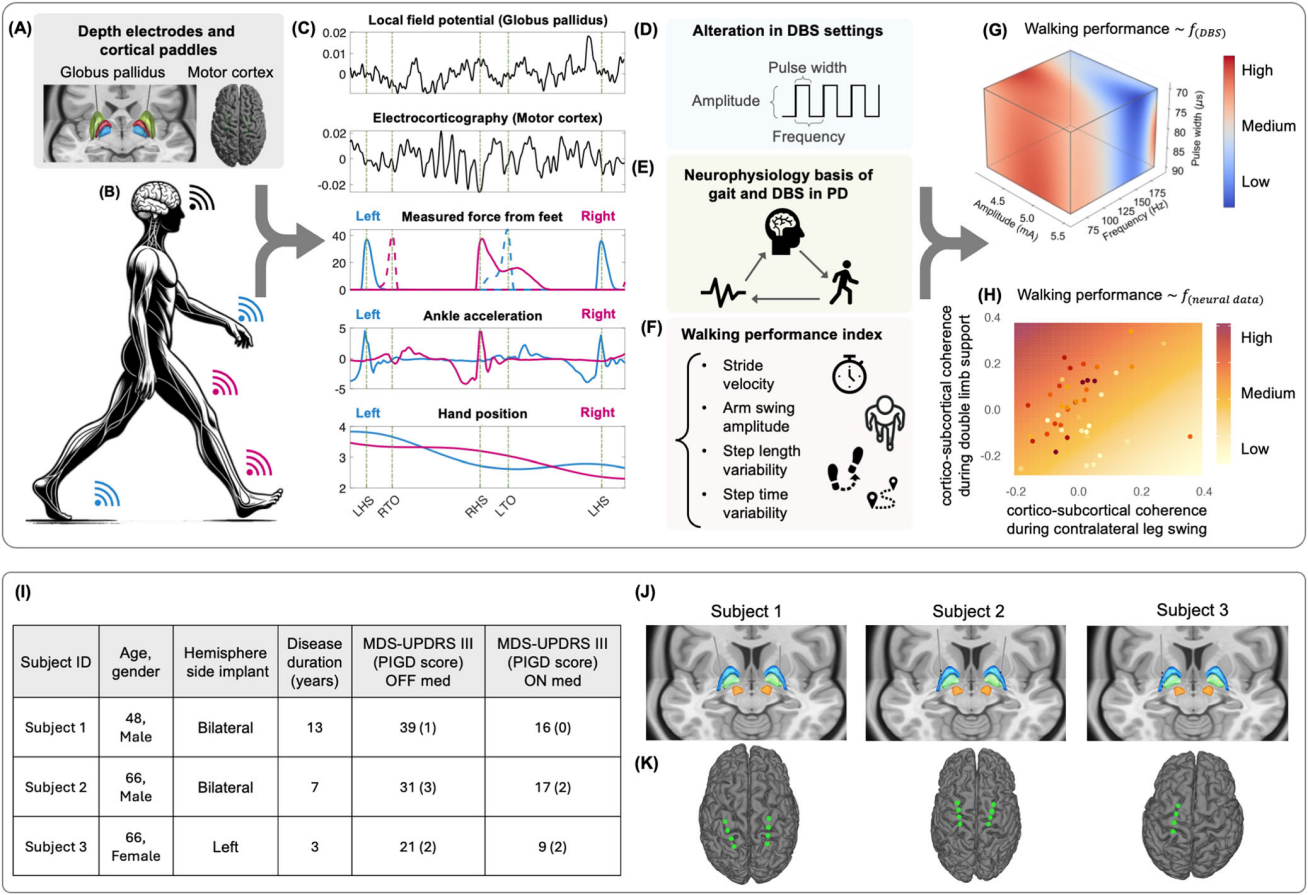
Workflow overview, patient characteristics, lead reconstruction, and electrode placement. **A** illustrates the DBS depth electrodes implanted along with the cortical paddles placed to stimulate electrical impulses and capture neural activity. **B** depicts a subject with Parkinson’s disease (PD) wearing a combination of sensors (i.e., Trigno system (Delsys) and MVN Analyze (Xsens)) to capture various gait kinematics. **C** shows a sample of data collected during overground walking. The first two subpanels display neural data from the Globus pallidus and motor cortex. Next are the gait kinematics, such as force sensors and ankle acceleration to capture gait events and spatiotemporal measurements to monitor body movements like arm swing amplitudes. **D** outlines the components of stimulation parameters altered in our experiments. **E** summarizes the goals of our analysis, which aim to understand the links between DBS settings, neurophysiological characteristics, and gait functions in PD. **F** Details the metrics employed to assess the impacts of DBS settings on walking performance, including variability in step length and step time, stride velocity, and arm swing amplitude. **G** presents sample results from our data-driven approach, mapping the relationship between DBS setting parameters and walking performance, where deeper red colors indicate higher walking performance. **H** exemplifies the outcome of our study in uncovering the neurophysiological bases of DBS settings associated with improved walking performance, where the walking performance is a function of the cortical-subcortical coherence during different phases of the gait cycle. **I** shows patient characteristics. MDS-UPDRS-III Movement Disorders Society Unified Parkinson’s Disease Rating Scale; Part III: motor domain, PIGD Posture Instability Gait Disorder (subscore from items 3.9: arising from the chair, 3.10: gait; 3.11: freezing, 3.12: postural instability, and 3.13: posture. Panels **J** and **K** display depth pallidal electrodes and cortical paddles, respectively.

Our study addresses these challenges by evaluating and modeling the effects of different DBS setting parameters on gait metrics and cortico-basal ganglia neurophysiology. We first developed a walking performance index (WPI) that integrates key gait kinematics to objectively assess and track gait performance across different DBS configurations. We then applied a Bayesian optimization method for modeling predicted walking performance based on the DBS settings for each subject, which efficiently delineates these relationships with limited trials. The data-driven model approach provided a systematic pipeline to identify effective person-specific DBS settings for gait improvement. Finally, by studying how DBS influences the pallidal and motor cortical network, we identified neurophysiological biomarkers associated with improved walking performance, which can further guide programming in the future (Fig. 1). These findings significantly contribute to our understanding of DBS’s impact on gait disorders and support the development of personalized data-driven models of DBS parameter optimization that refine neuronal activity and enhance gait outcomes. This methodology could be adapted to address a variety of other symptoms within PD, potentially offering a framework for similar approaches in other circuit-based disorders.

## Results

### Patient characteristics and electrode placement

Three patients with PD (PwP) (one female, two males) undergoing DBS implantation for motor fluctuations were recruited and implanted with a bidirectional investigational device (Summit RC+S, Medtronic, Inc.). Two participants were implanted bilaterally, while one received a unilateral implant in the left hemisphere. All subjects had gait disturbances (Fig. 1I). Gait disturbances in our subjects were assessed using the Movement Disorder Society’s UPDRS Part III (MDS-UPDRS-III), focusing on the motor symptoms associated with PD. Additionally, the posture instability and gait disorder (PIGD) sub-score provided a more specific evaluation of gait-related challenges, including difficulties with arising from a chair, walking, freezing episodes, postural instability, and overall posture^56^. All subjects were implanted with quadripolar DBS leads targeting the globus pallidus (GP) and quadripolar electrocorticography (ECoG) paddles placed in the subdural space over the motor cortical area. Figure 1J, K show the reconstructed lead locations from both the GP and motor cortex areas. The cortical electrode strip was positioned medial to the “hand knob” area of the primary motor (M1) cortex, which we have shown previously to demonstrate frequency-specific leg-movement related changes^57^. In Subject 1, the electrode primarily records from the somatosensory (S1) and M1 cortices, while in subjects 2 and 3, it spans both the M1 and premotor (PM) cortices. These regions exhibit somatotopic organization, where different body parts are represented in specific cortical areas, allowing for precise motor control and sensory processing. Each cortical paddle and the GP DBS lead in the same brain hemisphere were connected to a bidirectional Summit RC+S neural stimulator. This device is used to deliver electrical impulses and can chronically stream high-resolution time-domain data^58^.

### Walking performance index reveals changes in gait functions under different DBS stimulation parameters

**Fig. 2:**
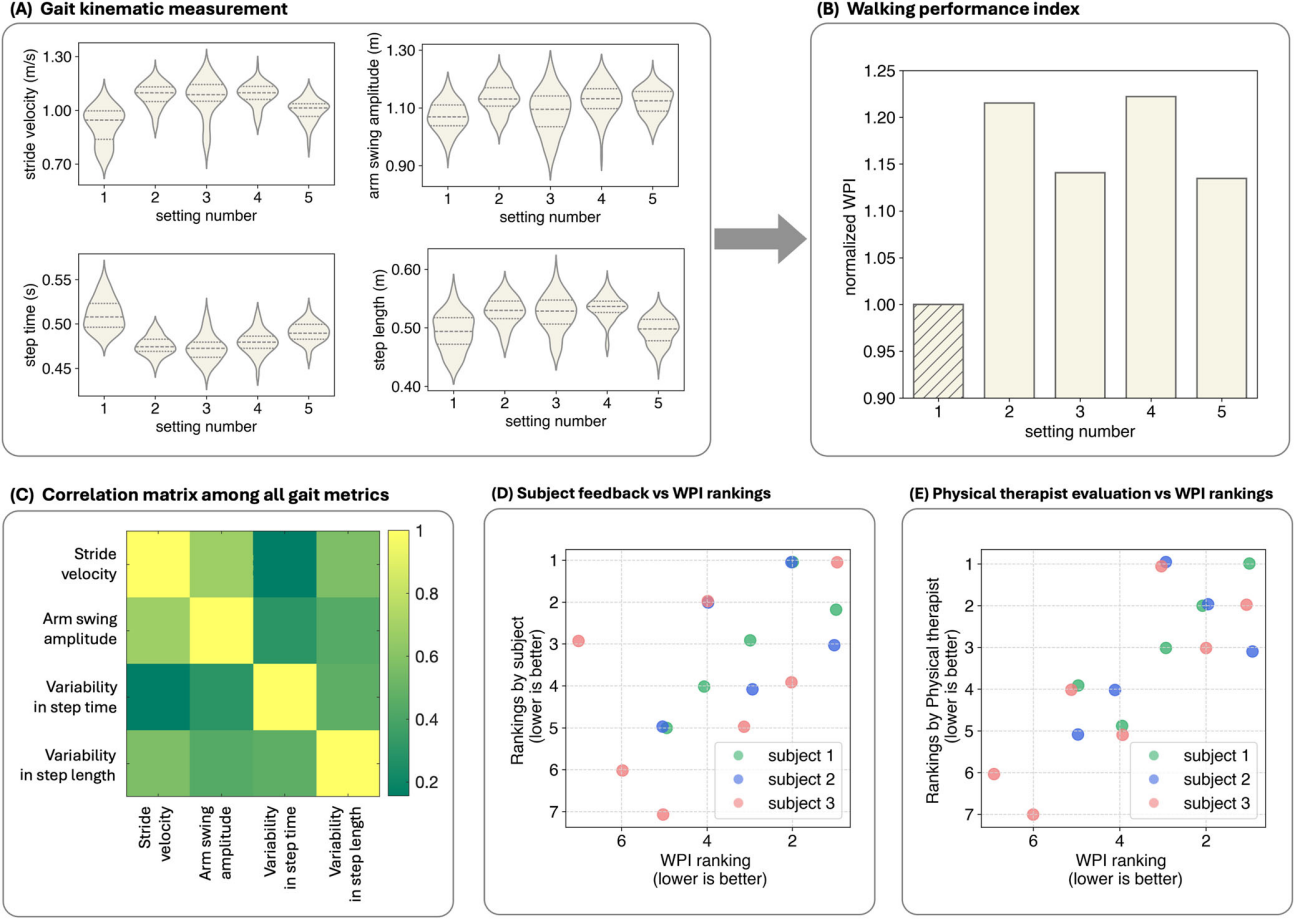
Walking performance development. **A** shows an example of the variations in gait kinematics in response to different DBS settings. **B** displays the corresponding fluctuations in the Walking Performance Index (WPI). The column with the hatched pattern represents the clinically optimized DBS settings tested at each visit. Heatmap in (**C**) shows the pairwise Pearson correlation coefficients among all gait metrics and the WPI. The matrix visualizes the degree of linear relationships between variables, with color intensity representing the strength of the correlations: colors range from green (lower values) to yellow (higher values). The low off-diagonal values (green shades) indicate minimal multicollinearity among the metrics, confirming that each metric provides unique and independent information contributing to the WPI. **D** and **E** show the correlation between the WPI rankings and feedback received from the subject (**D**), and evaluations from the physical therapist (**E**). Green, blue, and red dots represent data from subjects 1, 2, and 3, respectively. The x-axis displays the rankings based on WPI scores, while the y-axis shows the rankings provided by the patients. In both axes, lower numbers indicate higher rankings, with a rank of 1 representing the best performance or preference.

To objectively evaluate overground walking metrics across all participants and assess their fluctuations in response to alterations in DBS settings, we developed a WPI, which comprises four kinematic parameters associated with common gait deviations in PwP (Fig. 2A, B): stride velocity (i.e., the average walking speed over a complete gait cycle), arm swing amplitudes, and variability in step length and step time. Higher WPI indicates quicker stride velocity, larger arm swing amplitudes, and lower variability in step length and step time. During each visit, we tested both clinically optimized settings (i.e., adjusted by each patient’s Movement Disorders neurologist) and several new DBS configurations by varying the stimulation frequency, amplitude, or pulse width within safe ranges. Patients performed overground walking in a 6 m loop while their neural data and gait kinematics were continuously recorded. Each setting was evaluated over 200 steps, excluding turns, with turns evenly distributed between left and right. Gait metrics were calculated from full-body inertial measurement unit (IMU) sensors that precisely capture gait kinematics. Longer walking distances are generally advantageous for capturing steady-state gait patterns by minimizing the effects of acceleration and deceleration. However, due to spatial constraints, participants completed a 6 m overground walk. To assess whether acceleration and deceleration phases influenced our data, we analyzed stride times by categorizing strides based on their sequence (see Supplementary Fig. 1). The analysis revealed no significant differences in stride times related to stride order across all participants. These findings indicate that the 6 m distance was sufficient to achieve consistent walking patterns, supporting its adequacy for evaluating overground walking performance within the scope of our study.

We assigned equal weights to each of the four gait parameters to ensure a balanced contribution from all variables and then normalized the WPI to that of their clinically optimized settings. To validate the independence of each metric, we conducted a Variance Inflation Factor (VIF) analysis, which revealed VIF values ranging from 1.01 to 3.31, all below the commonly accepted threshold of 5. Additionally, a sensitivity analysis was performed by applying a ±10% change to each metric, demonstrating varying levels of impact on the WPI across individuals. The detailed correlation matrices, VIF results, and sensitivity analysis are presented in Supplementary Fig. 2.

For each subject, we tested up to three amplitudes: clinical amplitude, 25–30% reduction from clinical and their higher limits (i.e., 4.1–5.5 mA for Subject 1, 2.8–5 mA for Subject 2, and 3.5–4.9 mA for Subject 3). In addition to the clinical frequency, we tested 60 Hz as well as a higher frequency setting (i.e., 60/190 Hz for Subject 1, 60/180 Hz for Subject 2, and 60/190 Hz for Subject 3). We also tested two pulse widths: clinical values and their limits (i.e., 70/90 μs for Subject 1, 60/70 μs for Subject 2, and 60/80 μs for Subject 3). All these ranges were within the safety ranges defined by each patient’s neurologist. The details and range of DBS setting parameters are presented in the bottom panels of Supplementary Figs. 3–5. The intervals between visits for all subjects averaged 64 days (±45 days), with a range of 12–156 days. As a result of changes in these DBS setting parameters, we observed significant modifications in subjects’ gait kinematics and WPI. Table 1 summarizes the minimum and maximum values of the gait metrics and WPI for each subject.

**Table 1:** Changes in gait metrics for each subject

Subject ID	Gait Metric	Minimum	Maximum	Figure
		Value	Value	Reference
Subject 1	Stride velocity (m/s)	0.5444	1.7217	Supplementary Fig. 3A
	Arm swing amplitude (m)	0.6571	1.5453	Supplementary Fig. 3B
	Step time (s)	0.3983	0.6075	Supplementary Fig. 3C
	Step length (m)	0.3511	0.8731	Supplementary Fig. 3D
	WPI	0.4660	1.5597	Supplementary Fig. 3E
Subject 2	Stride velocity (m/s)	0.4738	1.2038	Supplementary Fig. 4A
	Arm swing amplitude (m)	0.3631	1.5121	Supplementary Fig. 4B
	Step time (s)	0.3105	0.7705	Supplementary Fig. 4C
	Step length (m)	0.2392	0.7897	Supplementary Fig. 4D
	WPI	0.2656	1.3205	Supplementary Fig. 4E
Subject 3	Stride velocity (m/s)	0.6345	1.3132	Supplementary Fig. 5A
	Arm swing amplitude (m)	0.7215	1.5809	Supplementary Fig. 5B
	Step time (s)	0.4339	0.5689	Supplementary Fig. 5C
	Step length (m)	0.3605	0.6166	Supplementary Fig. 5D
	WPI	0.4352	1.4185	Supplementary Fig. 5E

Furthermore, participants were blinded to the changes in DBS settings during the trials. After each trial, their subjective feedback was collected to assess their perception of walking performance under different stimulation settings and compared to that of a blinded physical therapist. We show a good correlation between the WPI and the rankings provided by both the subject and the physical therapist (Figs. 2D, E). This demonstrates the validity of the WPI to capture gait changes.

### Data-driven model identifies optimal DBS settings to improve gait in PwP

**Fig. 3:**
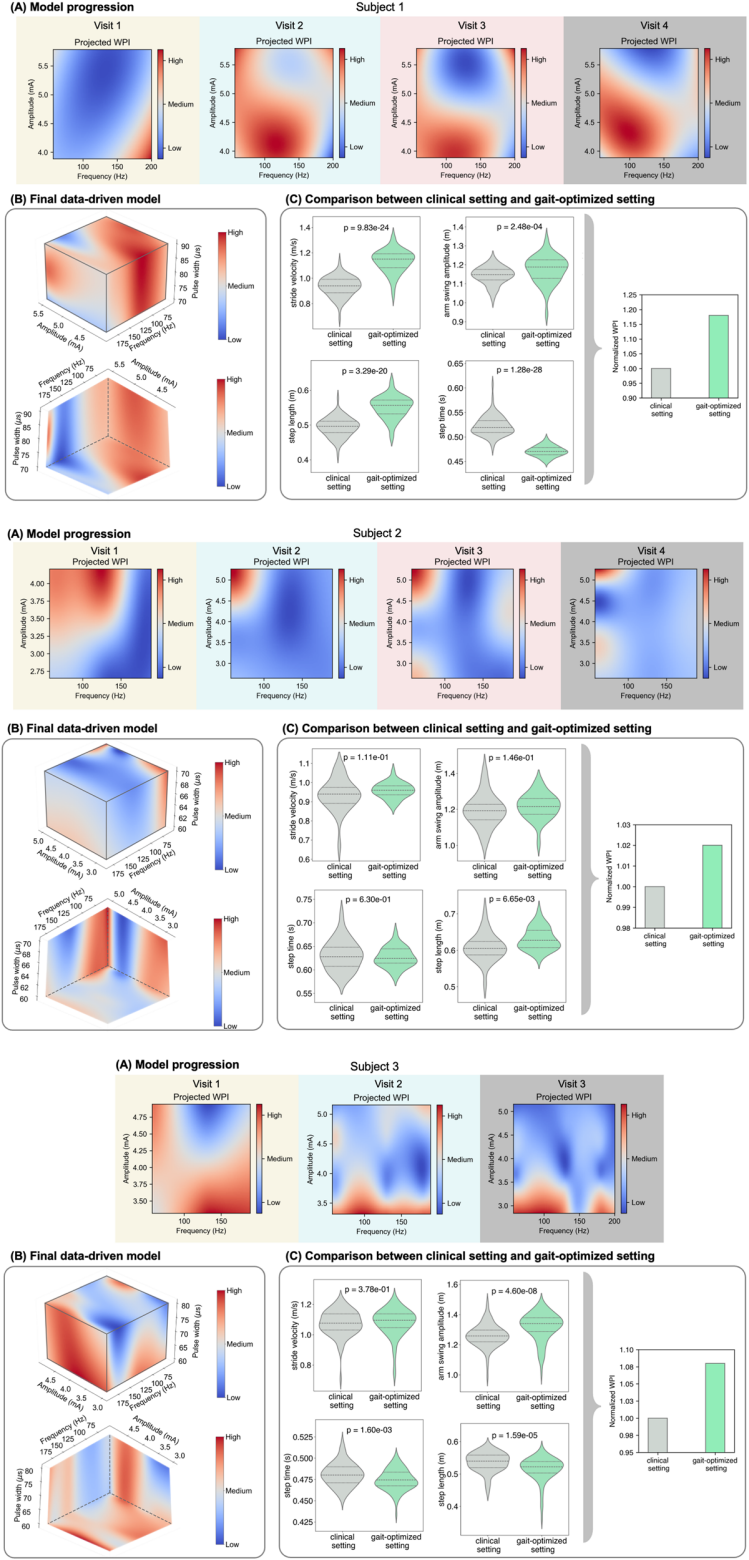
Data-driven modeling and optimization of DBS settings. For each subject, **A** depicts the progression of the Gaussian Process Regression (GPR) model in 2-D planes, updated with data from each visit. **B** showcases the final GPR model, mapping the relationship between DBS settings (amplitude, frequency, and pulse width) and WPI levels. The two perspectives illustrate the influence of amplitude, frequency, and pulse width on WPI levels, with red indicating higher walking performance and blue indicating lower WPI levels. **C** Compares WPI levels and associated gait kinematics under the clinical setting versus the gait-optimized setting derived from the GPR model.

To predict optimal DBS settings to enhance gait functions in PwP, we employed a data-driven Gaussian process regressor (GPR) to map the relationship between the DBS settings (input) and the WPI (output) for each subject (see Fig. 3). GPR is particularly appropriate for this application because it can effectively interpolate data from a limited number of tested settings to predict outcomes across a defined safe range of the parameter space. This approach allows for the efficient identification of optimal settings by leveraging the relationships between DBS parameters and gait performance, even when only a few settings have been experimentally tested. The GPR’s ability to model complex, nonlinear relationships and provide uncertainty estimates makes it beneficial for optimizing personalized DBS settings. We utilized the GPR after testing an average of 11 stimulation configurations to predict the next groups of settings to be tested. At each subsequent visit, we tested these model-predicted settings (best and worst) along with the clinically optimized setting. The resulting data were incorporated back into the GPR for further refinement.

The GPR model was able to predict and identify the optimal settings after testing several DBS configurations (i.e., 13 for Subject 1, 20 for Subject 2, and 11 for Subject 3). Across all patients, the gait-optimized settings predicted by the model led to an overall improvement in WPI, with specific degrees of improvement varying by patient. Panels (A) in Fig. 3 demonstrate the progression of the model between the DBS amplitude and frequency and WPI in 2D planes. In these plots, a deeper red color is associated with settings linked to higher walking performance levels. It illustrates how including the data from each visit would contribute to the changes in model dynamics, with the final model shown in Panels (B) of Fig. 3. Each patient exhibited a unique gait-optimized setting. For Subject 1, the optimal setting of 5.1 mA, 60 Hz, and 90 μs showed a significant 18% improvement in WPI over the clinical setting of 5.5 mA, 150 Hz, and 90 μs (Fig. 3B). Subject 2’s gait was optimized at 4.0 mA, 60 Hz, and 60 μs, compared to the clinical setting of 4.0 mA, 130 Hz, and 60 μs, leading to a modest 2% improvement in WPI (Fig. 3B). For Subject 3, the model predicted that a setting of 4.2 mA, 180 Hz, and 80 μs would outperform the clinically optimized setting of 3.9 mA, 145 Hz, and 60 μs. Testing this prediction resulted in an 8% improvement in WPI (Fig. 3B). Moreover, applying a linear mixed-effects model to analyze the relationship between subject feedback and WPI rankings, we found a significant positive relationship (*β* = 0.58, *p* = 0.005), indicating that higher WPI values tended to align with higher subjective ratings (Fig. 2D).

For comparisons of gait parameters under clinical DBS settings and gait-optimized DBS settings (Fig. 3C), each subject’s data were analyzed separately. There were notable unique variations in individual gait parameters across the subjects (Panels (C) in Fig. 3). Subject 1 exhibited the most pronounced improvements, with stride velocity increasing by 21.08% (Wilcoxon signed-rank test, *p* < 0.0001) and arm swing amplitude by 2.71% (*p* < 0.0005). This subject also demonstrated a noticeable 46.83% reduction in step time variability and an 18.93% increase in step length variability (Fig. 3C).

Subject 2 showed a 2.34% increase in stride velocity (*p* = 0.238) and a 1.98% increase in arm swing amplitude (*p* = 0.336). This subject experienced a substantial reduction in gait variability, with step time variability decreasing by 34.96% and step length variability by 19.96%. In Subject 3, stride velocity increased by 0.40% (*p* = 0.959), arm swing amplitude improved by 4.70% (*p* < 0.0002), and step time variability decreased by 11.22%. However, step length variability increased by 22.34% in Subject 3 (Fig. 3C).

In Subject 1, after identifying and validating the gait-optimized DBS settings, we presented these parameters to their movement disorders neurologist for clinical confirmation. After evaluating the optimized settings, the patient’s device was programmed with a new group with gait-optimized settings. The patient was given the option to switch to this gait-optimized setting during longer walks and return to the clinical setting designed to manage their other symptoms as needed. An analysis of the device logs revealed that over a period of 64 days, the patient voluntarily used the gait-optimized setting for an average of 4 h and 37 min per day (see Supplementary Fig. 6). These findings demonstrate the real-world applicability and efficacy of our proposed pipeline for identifying and implementing personalized DBS settings to improve gait functions, further validating its utility beyond controlled clinical environments.

Importantly, the gait-optimized DBS settings are designed to enhance gait functions without replacing clinical settings. To assess the long-term effects of gait-optimized DBS settings, we conducted additional experiments in which participants maintained their identified DBS parameters for a minimum of 1 h during daily activities. UPDRS and PIGD scores were evaluated after this period. The results demonstrated no significant adverse effects on overall UPDRS and PIGD scores, indicating that the gait-optimized settings did not negatively impact other aspects of motor function. Specifically, the UPDRS (PIGD) scores for the patients were as follows: Subject 1: 36 (7), Subject 2: 30 (4), and Subject 3: 13 (2). These findings suggest that gait-optimized DBS settings can enhance walking performance without compromising other motor functions.

### Identification of neural spectral changes across the gait cycle

**Fig. 4:**
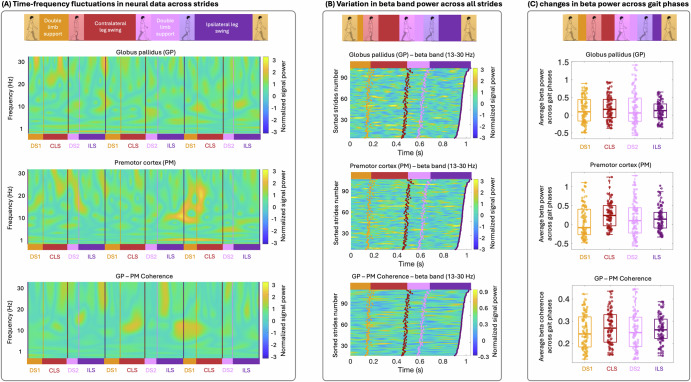
Neural data analysis and feature extraction. **A** demonstrates a sample of continuous wavelet transformation of the neural data across three complete gait cycles from the GP (top), PM (middle), and GP-PM coherence (bottom) sub-panel from the left brain hemisphere, respectively. Each row shows the normalized values of a single frequency, initiated at the left heel strike (purple lines). Within each row, purple lines denote the left heel strike (LHS), orange lines denote the right toe-off (RTO), red lines denote the right heel strike (RHS), and pink lines denote the left toe-off (LTO) moments. **B** demonstrates the average levels of signal power and coherence within the beta band (12–30 Hz) across all gait cycles, sorted by stride time and initiated at the LHS. Within each row, orange dots denote the RTO, red dots denote the RHS, and pink dots denote the LTO moments. All gait cycles conclude with the subsequent LHS (purple dots). Gait phases include DS1 (double limb support 1) from LHS to RTO, CLS (contralateral leg swing) from RTO to RHS, DS2 (double limb support 2) from RHS to LTO, and ILS (ipsilateral leg swing) from LTO to LHS. Boxplots in (**C**) represent average levels of the beta band during different phases of the gait cycle. Orange, red, pink, and purple colors, in turn, show the DS1, CLS, DS2, and ILS phases of the gait cycle.

The neurophysiological investigation of our study focused on identifying the average levels of signal power and coherence within distinct phases of the gait cycle and across canonical frequency bands. These features were extracted to capture the dynamic changes in neural activity and connectivity as patients moved through different stages of the gait cycle. An example of this analysis is presented in Panel (A) of Fig. 4. Each gait cycle begins with the left heel strike (LHS) (purple line), followed by the right toe off (RTO) (orange line), right heel strike (RHS) (red line), and left toe off (LTO) (pink line), culminating in the next LHS. The phases of the gait cycle are defined as follows: the first double limb support period (DS1: LHS-RTO), contralateral leg swing (CLS: RTO-RHS), second double limb support period (DS2: RHS-LTO), and ipsilateral leg swing (ILS: LTO-LHS). As an example of characterizing the changes within canonical frequency bands across the gait cycle, we present the spectral power and coherence within the beta band (12–30 Hz) across all gait cycles (Fig. 4B). Each row represents the average signal power or coherence within the beta band for a single gait cycle, sorted by stride time and initiated at LHS. This analysis revealed dynamic fluctuations in neural activity and coherence throughout the different phases of the gait cycle within each canonical frequency band (Fig. 4C). These findings provide essential insights into how DBS settings modulate neural spectral dynamics across different phases of the gait cycle, thereby advancing our understanding of the neural mechanisms driving gait improvements. Consequently, this knowledge facilitates the electrophysiology-based optimization of DBS parameters to enhance gait functions.

### Personalized neural biomarkers associated with gait improvements

To explore the relationship between neural spectral features and walking performance, we employed ordinary linear regression models on a single-subject basis. For each contact, separate models were fitted using the average power levels within canonical frequency bands across distinct phases of the gait cycle. In each model, all the neural signatures across all gait phases were included as predictors to determine which phase was most strongly associated with the WPI. To control for multiple comparisons, *p*-values were adjusted using the Benjamini-Hochberg (BH) method. Table 2 summarizes the personalized significant correlations (i.e., *p* < 0.05) observed across cortical and subcortical regions, as well as cortico-subcortical coherence, based on aggregated data across all stimulation settings.

**Table 2:** Summary of person-specific power and coherence neural biomarkers

Spectral power								
Subject ID	Recording site	Frequency band	Gait phase	Estimate	SE	*R*^2^	*p*-value	Adjusted *p*-value
Subject 1	S1	beta	ILS	1.400	0.483	0.316	0.0086	0.021
	GP	beta	DS1	1.783	0.652	0.387	0.0123	0.030
Subject 3	M1	theta	CLS	0.230	0.078	0.577	0.0094	0.023

Wavelet coherence								
Subject ID	Coherence	Frequency band	Gait pahse	Estimate	SE	*R*^2^	*p*-value	Adjusted *p*-value
Subject 1	GP–S1	theta	ILS	–10.782	3.95	0.30	0.0126	0.031
Subject 2	GP–M1	beta	DS2	–1.901	0.66	0.361	0.0120	0.028
Subject 3	GP–PM	beta	ILS	–4.220	1.35	0.392	0.0066	0.016
	M1–PM	theta	DS1	–3.686	1.25	0.443	0.0096	0.024

*S1* Somatosensory, *GP* Globus Pallidus, *M1* Primary motor, *PM* Premotor, *ILS* Ipsilateral leg swing, *CLS* Contralateral leg swing, *DS1* Double limb support following the ILS, *DS2* Double limb support following the CLS.

In Subject 1, S1 beta power (12–30 Hz) during the ipsilateral leg swing phase was positively correlated with walking performance (*p* < 0.01); this association remained robust after BH adjustment (*β* = 1.40 ± 0.48, adjusted *p* ≈ 0.021). Additionally, GP beta power during double limb support preceding the contralateral leg swing showed a positive association with walking performance (*β* = 1.78 ± 0.65, adjusted *p* ≈ 0.031), while GP-S1 theta coherence (4–8 Hz) during the ipsilateral leg swing was negatively correlated (*β* = −10.78 ± 3.95, adjusted *p* ≈ 0.032). In Subject 2, although no spectral power measures reached significance, GP-M1 beta coherence during double limb support (post-contralateral leg swing) was negatively correlated with WPI (*β* = −1.90 ± 0.67, adjusted *p* ≈ 0.028). In Subject 3, M1 beta power during the contralateral leg swing was positively associated with walking performance (*β* = 0.23 ± 0.08, adjusted *p* ≈ 0.024); furthermore, both GP-PM beta coherence during the ipsilateral leg swing (*β* = −4.22 ± 1.35, adjusted *p* ≈ 0.016) and M1-PM theta coherence during double limb support following the ipsilateral leg swing (*β* = −3.69 ± 1.25, adjusted *p* ≈ 0.024) were negatively correlated with performance. Additional details on these person-specific features are provided in Table 2.

### Shared neural spectral features correlate with walking performance across subjects

**Fig. 5:**
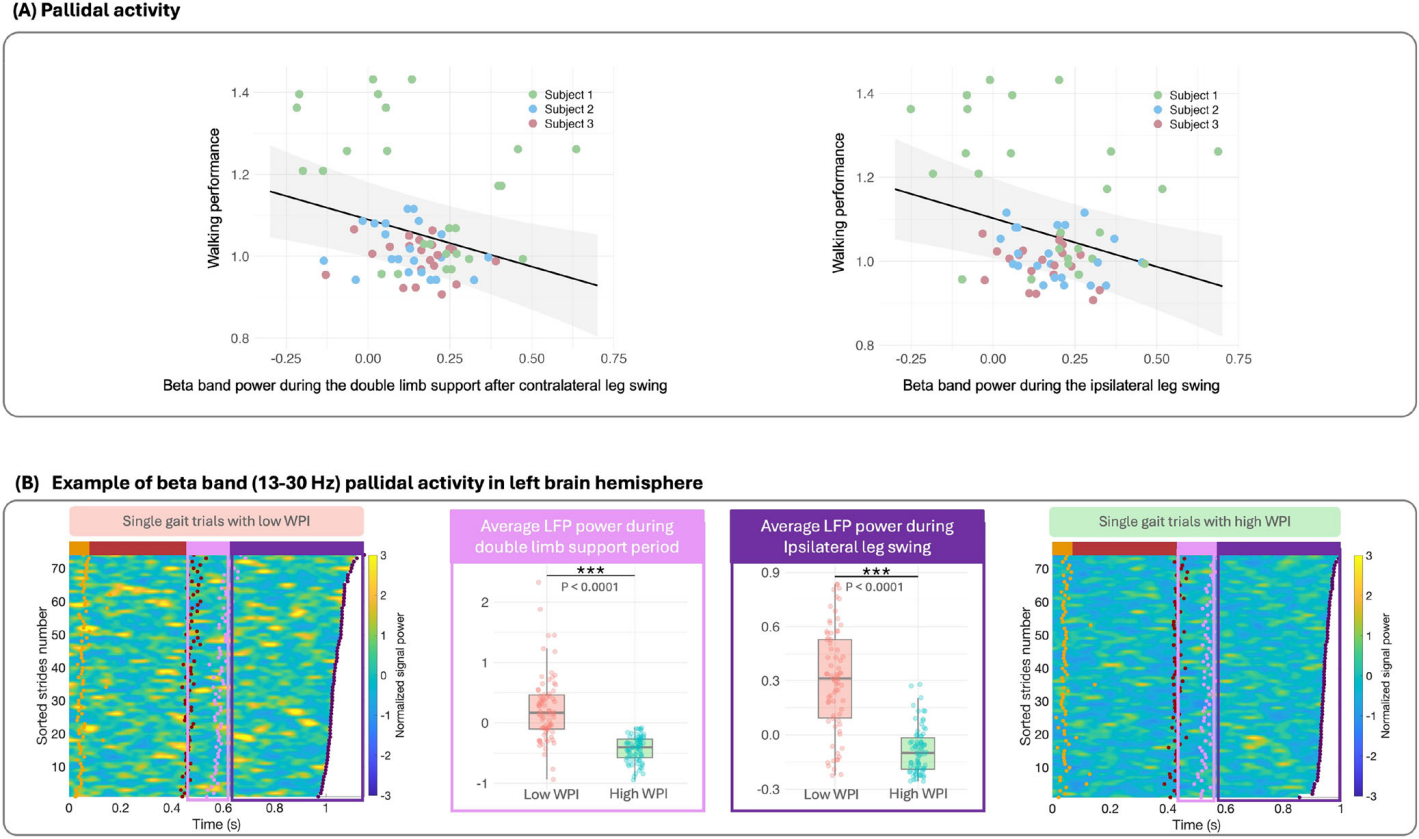
Correlation analysis of neural signal characteristics and walking performance. **A** presents the relationship between changes in LFP signal power in the pallidal area and walking performance. Left and right sub-panels, in turn, show the relationship between the walking performance and the beta band power during the double limb support period after the contralateral leg swing and the ipsilateral leg swing, respectively. **B** exemplifies the comparison of the neural signal variations from the left brain hemisphere between the sessions with low and high WPI across all single gait cycles. **B** illustrates the beta band power of LFP signal power in the pallidal area. The heatmaps on the left and right correspond to the lowest and highest walking performance, respectively. Each row represents a single gait trial, sorted by stride time and initiated at the left heel strike. Orange dots denote the right toe-off, red dots indicate the right heel strike, and purple dots represent the left toe-off. All gait cycles conclude with the subsequent left heel strike. Middle sub-panels compare signal power during the specific gait phases of interest (i.e., double limb support period and ipsilateral leg swing in (**B**), contrasting sessions with the lowest WPI (shown in red boxplots) and the highest WPI (shown in green boxplots).

To identify shared spectral features associated with improvements in gait performance across individuals, we used a linear mixed model to evaluate neural biomarkers of walking performance. Given the exploratory nature of our analysis, we focused on assessing correlations between individual neural features and walking performance, rather than constructing a comprehensive model incorporating all potential features simultaneously. By analyzing each feature separately, we aimed to identify consistent neural biomarkers across all participants that correlate with walking performance. This model included fixed effects for each neural feature and each hemisphere, as well as a random intercept for each individual. We extracted and averaged spectral power across all gait cycle epochs and assessed the correlation with the average levels of walking performance across all participants, covering multiple visits and different DBS settings. We observed a significant negative correlation between the beta band of the LFP signal power in the pallidal region and the increase in overall walking performance (Fig. 5A, B). This significant correlation was primarily seen in the beta band power during the double limb support following the contralateral leg swing and the ipsilateral leg swing phases of the gait cycle, constituting the contralateral leg stance phase. Specifically, in the double limb support phase, beta band power was found to be associated with walking performance, with higher performance associated with lower beta band power (*β* = −0.22 ± 0.078, *t*(66) = −2.93, *p* = 0.0046). Including hemisphere as an interaction term did not improve model fit (Δ*AIC* = 1.2; interaction *p* = 0.37), and baseline WPI did not significantly differ by hemisphere (*p* = 0.73). Thus, lower GP beta power during the double limb support following the contralateral leg swing is associated with higher walking performance to the same extent in both hemispheres. Similarly, during the ipsilateral leg swing phase, beta band power from the pallidal area was found to be negatively associated with walking performance (*β* = −0.23 ± 0.08, *t*(66) = −2.89, *p* = 0.0050). Including hemisphere as an interaction term did not improve model fit (Δ*AIC* = 1.0; interaction *p* = 0.30), and baseline WPI did not significantly differ by hemisphere (*p* = 0.65). Therefore, lower GP beta power during the ipsilateral leg swing phase corresponds to higher walking performance to the same extent in both hemispheres. After BH correction, both beta-band features remain significant: double-limb support *χ*^2^ ≈ 12.2, adjusted *p* = 0.0183; ipsilateral leg swing *χ*^2^ ≈ 12.0, adjusted *p* = 0.0183.

To assess the assumptions of normality and homoscedasticity for all linear mixed effects models, Shapiro-Wilk tests showed no significant deviation from normality (*p* = 0.337 for the beta band power during the double limb support following the contralateral leg swing phase, and *p* = 0.279 for ipsilateral leg swing phase), and skewness and kurtosis values were within acceptable ranges (skewness < 0.39; kurtosis < 3.6). Visual inspection of Q–Q plots and residual-versus-fitted plots supported these findings, with no evidence of major deviation from model assumptions. These findings suggest that a reduction in beta band power in the pallidal region during the contralateral leg stance phase is a critical neural marker for improved gait outcomes.

To further illustrate these neurophysiological characteristics and their relationship with walking performance, we compared neural activity between sessions with the lowest and highest WPI levels in Subject 1 (Fig. 5B). This Figure highlights key differences in beta-band power in the pallidal area from the left hemisphere. The heatmap on the left side of Fig. 5B displays the neural activity associated with the lowest walking performance, while that on the right corresponds to the highest performance. The middle sub-panels provide a more focused comparison of signal power during key phases: double limb support after the contralateral leg swing and ipsilateral leg swing in Fig. 5B, contrasting sessions with the lowest WPI (represented by red boxplots) and the highest WPI (represented by green boxplots). These visualizations underscore the distinct neural dynamics that differentiate optimal gait performance from less effective patterns.

## Discussion

We developed a data-driven pipeline to identify optimized DBS setting parameters for enhancing gait in patients with PD (PwP). Our research is one of the first to systematically identify optimized DBS settings (i.e., amplitude, frequency, and pulse width) with a personalized data-driven approach targeting several spatiotemporal aspects of gait. The proposed model maps the relationship between DBS settings and walking performance across a wide range of parameter values, allowing us to predict and identify optimal stimulation configurations beyond the discrete settings initially tested, thereby significantly enhancing walking performance. Additionally, we identified neural biomarkers associated with improved walking performance, revealing both consistent characteristics across patients and features specific to each individual. The proposed pipeline links DBS configurations, underlying neural connectivity, and gait kinematics. Our results have several implications which are discussed below.

In this study, we developed an objective WPI based on kinematic measures to accurately assess the effectiveness of our approach. The WPI represents core aspects of gait often impaired in PwP, which can lead to significant functional limitations and increased fall risk^2,4,26–30,42,59^. Stride velocity indicates overall mobility, with reduced speed being a common symptom of PwP^29,32^. Arm swing amplitude reflects the coordinated movement necessary for balance^30^, while variability in step length and time provides insight into gait consistency, with greater variability often linked to instability and fall risk^26^. By combining these metrics, the WPI offers a more comprehensive assessment of gait, addressing multiple dimensions of motor function affected by PD. The choice of equal weighting in the WPI aimed to ensure a balanced contribution from all gait parameters, preventing any single metric from disproportionately influencing the overall score. While our analysis suggested that each gait metric contributes uniquely to the WPI, further examination revealed some degree of correlation between certain parameters, notably stride velocity and arm swing amplitude (see Supplementary Fig. 2). This correlation is inherent due to the biomechanical interdependence of these metrics, as variations in stride velocity naturally influence arm swing amplitude during walking^30,60^. Despite these moderate correlations, the VIF analysis confirmed low multicollinearity, indicating that each metric still provides distinct and valuable information for the WPI. Moreover, the sensitivity analysis highlighted that the extent of each metric’s contribution to the WPI varies among individuals, reflecting the diversity in individual walking styles. It is also worth mentioning that alternative weighting schemes could affect the sensitivity of the WPI to specific aspects of gait dysfunction. Future research might explore differential weighting based on the relative impact of each parameter on functional outcomes or patient quality of life.

The WPI’s broad evaluation of gait characteristics allows for a more robust assessment of DBS settings and their effect on walking. Our results confirmed that changes in DBS settings were effectively captured by the WPI, aligning with patient and clinician evaluations during each visit. This validation supports that the WPI is an effective metric for assessing and targeting gait improvements in PwP, potentially enhancing the precision of DBS programming. Future directions include developing automated systems for real-time gait analysis and integrating WPI with DBS programming software. Technologies such as gait mats^61,62^, wearable sensors^63–66^, and advanced motion capture systems^67,68^ could enable continuous and precise monitoring of gait, allowing for more accurate DBS adjustments. Several machine learning (ML) methodologies have been employed to analyze data from various gait measurement techniques, including wearable IMUs, force-sensitive gait mats, and advanced motion capture systems^67,69–72^. These approaches have enabled researchers to identify subtle gait anomalies, classify movement states, and predict clinical outcomes with greater accuracy^73–77^. By integrating such ML-driven analyses into the WPI framework, it becomes possible to refine the metric and tailor DBS programming even more effectively. This integration promises a more personalized, data-driven strategy for managing gait-related symptoms in PD, leveraging continuous sensor data and real-time feedback to guide DBS parameter adjustments. Further studies should also explore the WPI’s broader application across different populations and its potential to improve clinical outcomes in PwP and other movement disorders.

All participants conducted continuous overground walking trials on a 6 m walkway, during which neural activity and gait kinematics were concurrently recorded. While longer walking distances are typically preferred for capturing steady-state gait dynamics, our additional stride time variation analysis demonstrated that a 6 m walk was sufficient to achieve consistent walking patterns among participants. The lack of significant stride time differences between stride orders suggests that acceleration and deceleration phases did not substantially impact gait consistency within this distance (see Supplementary Fig. 1). This finding aligns with previous studies that have successfully utilized shorter walking distances without compromising the assessment of gait stability^78–80^. However, it is important to note that longer walks could provide more extensive data on gait dynamics and capture rare gait disturbances, such as festinating gait in PwP, which may not be fully observable within a confined space. Future research should consider incorporating longer walking distances when feasible to further validate and expand upon these findings.

Optimizing DBS settings to enhance gait in PwP is challenging due to the vast parameter space and the time-intensive process required during patient visits. Although programming DBS for motor symptoms such as bradykinesia and tremors generally yields quick results, gait disturbances are more complex and may take longer to respond, adding to the complexity of clinic visits. In addition, individual responses to DBS therapy vary significantly, necessitating a systematic, personalized approach to identify optimized settings. We hypothesized that the extensive DBS parameter space, combined with the complex nature of gait and individual variability, requires a personalized, data-driven approach. Unlike methods that compare discrete stimulation parameters, our aim was to establish a continuous map between DBS settings and walking performance. We employed a GPR to model these dynamics, using data from each visit with DBS settings as input and WPI as output. The GPR model, with its non-parametric nature, effectively captured the complex relationship between DBS settings and WPI by updating its predictions based on continuous variations in DBS settings.

Several studies have explored Bayesian optimization and related ML methodologies to streamline neuromodulation therapies^81–90^. For example, the authors in ref. ^91^ used a deep learning convolutional neural network on data from wearable inertial sensors to quantify Parkinsonian tremors and map its relationship to various DBS amplitude settings, thus allowing more accurate and personalized DBS programming for tremors management. Researchers in ref. ^86^ used Bayesian optimization to improve cognitive control, while authors in ref. ^87^ optimized a multi-objective function to maximize cortical evoked potentials and reduce side effects. Researchers introduced an adaptive dual controller using Bayesian optimization to identify the phasic and amplitude of stimulation to control simulated beta power^88^. Authors in ref. ^89^ identified optimal stimulation contact and amplitude to suppress tremors via Bayesian optimization. Researchers in ref. ^90^ proposed a semi-automated Bayesian optimization algorithm to tune stimulation frequency for handling rigidity in PD patients. Additionally, authors in ref. ^83^ verified the advantages of Bayesian optimization in tuning epidural spinal cord stimulation and identifying the frequency and pulse width of neurostimulation, and authors in ref. ^84^ applied Bayesian optimization for inhibiting hippocampal seizures in animal models. Researchers also used data-driven Bayesian-based methodologies to predict optimal DBS settings based on brain response patterns observed in functional magnetic resonance imaging data^85^. However, optimizing DBS parameters for gait remains a challenging task, and thus, we propose our GPR-based approach to identify optimal DBS settings that enhance gait functions in PD.

Our model incorporated several stimulation parameters—amplitude, frequency, and pulse width—as inputs, enabling us to understand the impact of each parameter in relation to the others on walking performance. One key advantage of this approach is its ability to explore the entire DBS parameter space (i.e., amplitude, frequency, and pulse width) within safety ranges defined by movement disorders neurologists, potentially deriving optimized settings beyond the parameters tested. Contact selection was kept constant and determined separately based on each patient’s movement disorder neurologist’s expertise, rather than being optimized by the model. In our GPR model, we employed a Matérn kernel that was chosen for its flexibility in modeling functions with varying degrees of smoothness, making it well-suited to capture the complex gait dynamics influenced by DBS settings. The initial length scales for amplitude, frequency, and pulse width control the rate at which correlation decays in the input space, thereby regulating the model’s smoothness and generalization capacity. By optimizing these hyperparameters based on the observed data, the GPR dynamically adjusts the prior to accurately capture both gradual and abrupt changes in gait parameters, enhancing predictive performance while preventing overfitting. The choice of the Matérn kernel and the optimization of its hyperparameters are crucial for the GPR’s ability to generalize from limited data points. Given the small sample size, the Matérn prior provides a balanced approach by offering sufficient flexibility to model complex patterns without introducing excessive variance. Recognizing the critical role of the prior in shaping model predictions, future research will explore alternative kernel functions and prior configurations to further enhance the accuracy of gait parameter modeling. Testing the identified settings further validated the model’s efficacy, resulting in significant improvements in walking performance. This approach could enhance the DBS programming process by better tracking the effects of changes in stimulation parameters on each spatiotemporal gait metric^92^.

An important finding of our work is that although patients’ gait-optimized settings are varied, there are consistent and convergent neural dynamics identified in these settings that are shared on a group level. To uncover spectral signatures linked to gait improvements across participants, we applied a linear mixed-effects model-incorporating fixed effects for each neural feature and hemisphere, plus a random intercept for each individual to assess walking performance biomarkers. Specifically, lower levels of pallidum beta-band activity during the contralateral stance phase were associated with improved walking performance. Previous studies have shown that beta power in basal ganglia regions correlates with PD off-symptom severity and that its suppression through dopaminergic therapy or DBS improves UPDRS scores^93–99^. Specifically during gait, reduced beta-band activity in the GPi is linked to improved performance^52^, and STN DBS can reduce high beta frequency power and bilateral oscillatory connectivity during gait^100,101^. This reduction in beta activity may reflect a general mechanism of release from motor inhibition, facilitating smoother, more coordinated, and faster movements^102^. Our findings deepen the understanding of the neural mechanisms underlying DBS-enhanced gait performance and emphasize the need to identify individualized DBS settings to enhance these patterns.

To assess the relationship between neural spectral features and walking performance, we also conducted individual-level linear regression analyses. We utilized signal power from pallidal LFPs and motor-cortical recordings via time-frequency analysis, and we used wavelet coherence between these regions to measure their functional connectivity during walking periods. Due to the nonstationary nature of the brain signals during the dynamic movement state of gait, we used wavelet coherence to capture how the cortex and basal ganglia coherence evolve over the period of time, rather than using either magnitude-squared coherence or imaginary coherence, which is more suitable for stationary signals. For each electrode contact and coherence pair, separate models were constructed using the average power recorded within canonical frequency bands across all distinct gait phases. In these models, every neural signature across the gait phases was included as a predictor to identify the phase most strongly associated with the WPI. We applied the BH correction to adjust the *p*-values for multiple comparisons (see Table 2 for additional details). Our analyses indicate that the neural signatures underlying enhanced walking performance are highly individualized. Specifically, the distinct clinical profiles of each subject-including differences in managed symptoms, personalized gait-optimized DBS settings, and unique responses to stimulation adjustments-appear to drive divergent patterns of neural oscillatory activity. In other words, as stimulation parameters are fine-tuned in a patient-specific manner, the resulting changes in neural activity emerge in unique ways that correlate with improved walking performance. This variability underscores the critical importance of personalized approaches for understanding and guiding therapeutic interventions.

Collectively, the neural biomarkers identified here—GP LFP power, cortical ECoG power, and cortico-subcortical coherence—underscore the complementary roles of basal-ganglia and cortical circuits in motor control^103^. Although the cortical strip in this study covers only a small portion of the motor cortex, the additional analyses conducted provide evidence that the recorded signals represent information associated with walking-related neural activity. The distinct signal power patterns observed during overground walking, as compared to isolated arm swings, indicate that our cortical recordings capture coordinated motor functions involving both lower and upper limbs^55,57^. This suggests that, despite the partial coverage, the cortical strip effectively monitors relevant aspects of motor cortical activity associated with walking. Nonetheless, we acknowledge that broader cortical coverage could offer a more complete representation of motor activity and potentially reveal additional neural dynamics related to walking. Future studies should consider using electrode arrays with wider coverage to further validate and expand upon our findings.

While our current DBS optimization pipeline still requires several visits by the patient, with the identification of key neurophysiological signatures associated with enhanced gait functions, we envision using advanced ML models in the future that can potentially predict stimulation parameters that boost these oscillations without the need for extensive tests. To further enhance the model and expedite the optimization process, reinforcement learning algorithms could dynamically adjust DBS settings in real-time based on continuous gait performance feedback, while deep neural networks could capture complex, nonlinear relationships between DBS parameters, neurophysiological dynamics, and gait performance, potentially improving prediction accuracy. Transfer learning could also utilize pre-trained models on similar datasets, reducing the data needed for training and accelerating optimization for new patients.

Our study highlights the importance of oscillatory activity during gait and the potential of these neural biomarkers to differentiate normal from impaired walking, enabling more effective therapy. Unlike well-established approaches that track biomarkers associated with other PD symptoms, such as beta suppression for akinesia and rigidity or gamma-band activity for dyskinesia, there is a notable lack of reliable biomarkers specifically linked to gait dysfunction in PwP. By identifying these network-level oscillations associated with gait improvement, we supply the missing link needed to facilitate the programming process and open avenues for developing adaptive, closed-loop DBS systems. These systems require a comprehensive understanding of how DBS parameters influence pathological neural oscillations and behavioral measures. In a human-in-the-loop framework, these oscillations could be tracked in real-time and used as feedback to update stimulation parameters, thereby modulating pathological rhythms and enhancing walking performance. Clarifying how specific DBS settings reshape neural activity and behavior not only advances DBS treatment of gait dysfunction in PwP but also holds significant potential for control-system-based approaches to treat motor symptoms across a wide range of neurodegenerative diseases.

Our study has limitations. The data-driven model’s performance was validated with a small number of participants, and future studies with larger populations are needed to confirm its efficacy in identifying optimized DBS settings with fewer trials. Moreover, the correlation observed between WPI rankings and subject feedback should also be verified in larger cohorts to ensure robust generalizability. We did not impose constraints on total energy delivery, which could be a future optimization target. Additionally, we relied on neurologists’ selections for GP stimulation contacts without exploring their effects in detail. Our analysis focused mainly on straight walking, suggesting that future models should incorporate factors like turning and gait initiation to improve treatment outcomes. Our exploratory analysis identified neural biomarkers associated with gait improvements at both the group and individual-specific levels. However, we recognize that the limited sample size and the investigative nature of this study may affect the generalizability of our findings. Future studies with larger cohorts and more extensive data collection are warranted to validate these neural biomarkers and to further interpret the neural mechanisms underlying gait improvements in response to DBS.

Our study systematically modeled the relationship between DBS setting parameters, underlying neural dynamics, and gait functions, demonstrating the efficacy of data-driven techniques in optimizing patient-specific DBS settings. By integrating key gait kinematics into our performance assessment, we developed a useful tool to target while enhancing gait functions through personalized DBS interventions. Our data-driven model effectively identified optimal DBS settings tailored to each patient, resulting in significant improvements in walking performance. The neural biomarkers we identified could be used to assess the impact of DBS settings on gait functions, providing insights for further optimization of stimulation parameters. While these preliminary findings highlight the potential of integrating gait metrics and neural biomarkers to guide stimulation parameters, we recognize that the small patient cohort limits the immediate generalizability of our results. Nonetheless, our approach enhances understanding of how DBS modulates gait and underscores the importance of personalized treatment strategies, with broader applicability to various neurodegenerative diseases. The identified neural biomarkers have the potential to be utilized in electrophysiology-based optimization of DBS settings targeting gait functions. Future work with larger, more diverse populations is needed to validate these findings and fully realize the promise of personalized DBS therapies.

## Methods

### Subjects’ recruitment, DBS surgery, and electrode localization

Three participants with PD and gait dysfunction (one female, two males, age range: 48–66 years, disease duration: 3–13 years) were recruited and implanted with Summit RC+S devices (two subjects received bilateral implants and one received unilateral implant), with subdural paddle electrodes over the motor cortex and DBS electrodes in the GP (Fig. 1J, K). Participants were enrolled in a clinical trial (ClinicalTrials.gov ID: NCT-03582891) at the University of California, San Francisco (UCSF), which was approved by the UCSF Institutional Review Board under the approval number 20-32847. The leads were attached to a research-grade, sensing-capable implantable pulse generator (Medtronic Summit RC+S model B35300R), which was positioned in a compartment over the pectoralis muscle on each side. Precise electrode localization was achieved through established image analysis pipelines for both depth and cortical electrodes. Post-implantation high-resolution CT images were briefly coregistered to preoperative T1-weighted 3T MRI using a rigid, linear affine transformation. The accuracy of coregistration was verified through visual inspection and, when necessary, refined using an additional brain shift correction routine to align subcortical anatomy. Electrode artifacts were then identified on CT and matched to known electrode geometry. Additionally, cortical electrodes were projected onto the MRI-rendered pial surface. More details regarding the surgical implantation and lead reconstruction can be found in refs. ^58,104–107^.

### Experiment procedures and data collection

For each subject, the DBS setting configuration was optimized before initiating the tasks. This optimization (i.e., their “clinical setting”) was completed by each patient’s Movement Disorders neurologist over a range of 1.5–3 months. All subjects were receiving their typical dose of Parkinsonian medication throughout data collection and follow-ups. In all participants, LFPs were recorded from the GP. ECoG was recorded from two pairs of contacts, identified via imaging-based reconstructions to capture activity from distinct cortical regions (Figs. 1J, K). In Subject 1, contacts 8 and 9 overlaid the somatosensory (S1) cortex, whereas in Subjects 2 and 3, they targeted the primary motor (M1) cortex. Contacts 10 and 11 were positioned over the M1 cortex in Subject 1 and over the PM cortex in Subjects 2 and 3. Neural data were acquired at a sampling rate of 500 Hz. Moreover, the Summit RC+S system’s built-in accelerometer, which synchronizes measurements with external sensors, collected accelerometry data at 64 Hz. We extracted and analyzed all the data using open-source code available in https://github.com/openmind-consortium/Analysis-rcs-data^108^.

During clinic visits, patients’ DBS settings were altered within safety ranges to examine their impacts on modulating gait functions. In response to each set of DBS settings, participants performed overground walking in a loop of 6 m with continuous streaming of their neural data and gait kinematics. Each setting was tested for 200 overground (non-turning) steps, with half the turns to the left and half to the right. Before each walking trial, the research team systematically changed the stimulation settings. These adjustments encompassed changes in stimulation amplitude, frequency, and pulse width. A minimum interval of 15 min was observed between each walking trial to allow a proper washout period. Following the completion of the overground walking tasks, subjects were instructed to sit and rest for 3 min. After this initial rest period, a member of the research team collected patient feedback using standardized questions covering the presence of any potential side effects, perceived effort with walking, gait symmetry, perceived balance and/or stability, and the overall feel of the setting compared to everyday function. Upon receiving their feedback, participants were asked to sit and rest as they received new sets of stimulation parameters. All trials were conducted in a randomized order with examiner and patient blinding.

We used two types of wireless technology to capture gait kinematics: Trigno system (Delsys) and MVN Analyze (Xsens). Delsys included two Avanti force-sensitive resistor (FSR) adapters, two Avanti goniometer adapters, and two Trigno surface electromyography (EMG) sensors with built-in accelerometers. EMG sensors were placed on the lower legs (bilateral soleus and tibialis anterior muscles) for precise muscle activity measurement. Each FSR adapter connects to four FSRs (model DC: F01, Delsys) positioned under the heel (calcaneus), big toe (hallux), base of the big toe (first metatarsal), and base of the pinky toe (fifth metatarsal). A digital goniometer (SG110/A) was placed next to the ankle bone (lateral malleolus) on each side to measure continuous ankle joint angles. Xsens uses 15 body-worn sensors to detect position and movement for full-body motion tracking^109^. Subjects were also video recorded with multiple camera views. The recordings were synchronized with the other recording devices to facilitate additional inspection and analyses.

### Integrating gait kinematics with walking performance index

This study focuses on straight-line overground walking; hence, turns were excluded from the analysis. Gait kinematics, including step length and step time duration, are derived utilizing Delsys and Xsens recordings. The initial detection of gait events necessary for our analysis is conducted using FSR sensors. However, in instances where gait peculiarities, such as toe walking, result in suboptimal signal strength, alternative methods (e.g., goniometer signal) were employed to ensure accurate identification of gait events. All gait events underwent visual inspection, with any inaccuracies manually adjusted. To analyze how walking performance is influenced by changes in DBS settings, we developed a WPI to capture various gait kinematics, including stride velocity, step length variability, step time variability, and arm swing amplitude. These metrics were selected based on their importance in the literature on PwP gait^26–32^:

1 $${{WPI}}=\frac{{{S{V}}}_{norm}+{{A{S}}}_{norm}+(1-{{VS{L}}}_{norm})+(1-{{VS{T}}}_{norm})}{4}$$

where *SV*_*norm*_ and *AS*_*norm*_ are the normalized values of stride velocity and arm swing amplitude, respectively, scaled so that higher values reflect better performance. *VSL*_*norm*_ and *VST*_*norm*_ are the normalized values of step length variability and step time variability, respectively, with higher values indicating greater variability. Thus, we use (1 − *VSL*_*norm*_) and (1 − *VST*_*norm*_) so that larger variability reduces the final WPI. During each visit, we normalized the WPI relative to the baseline levels observed in the clinical setting.

### Data-driven model development and identification of gait-optimized DBS setting

Leveraging data-driven methodologies, we modeled the relationship between distinct DBS settings as the input and the WPI as the output. Employing the Gaussian process regression (GPR) method, we created personalized maps detailing how DBS settings influence WPI for each participant, highlighting which stimulation settings could enhance WPI. GPR stands out as a flexible, non-parametric Bayesian approach, adept at capturing complex relationships between variables without presupposing the form of the underlying function^110^. This method excels in scenarios where the relationship’s exact nature is unknown or too complex to model with traditional parametric techniques. By integrating a potentially finite number of parameters, GPR allows the data to dictate the complexity of the model, adapting to the nuances of each individual’s response to DBS adjustments. Furthermore, GPR’s utility extends beyond passive analysis, facilitating active learning by optimizing input selection to maximize desired outcomes, making it an invaluable tool in the iterative process of identifying optimal DBS settings for enhancing gait performance. This approach aligns with previous studies across various domains, demonstrating the versatility and power of GPR in both understanding and optimizing human behavior and physiological responses. In our implementation, we adopt the Matérn kernel as the covariance function^111^, a choice driven by its flexibility and capability to capture the roughness of the functional relationship between DBS settings and WPI. The Matérn kernel is defined as:

2 $$k({x}_{i},{x}_{j})=\frac{1}{\Gamma (\nu ){2}^{\nu -1}}{\left(\frac{\sqrt{2\nu }}{l}d({x}_{i},{x}_{j})\right)}^{\nu }{K}_{\nu }\left(\frac{\sqrt{2\nu }}{l}d({x}_{i},{x}_{j})\right)$$

where *d*(. , . ) is the Euclidean distance, *K*_*ν*_(. ) is a modified Bessel function and *Γ*(. ) is the gamma function. The parameter *l* is associated with the length scale, while *ν* controls the smoothness of the resulting function. In this study, we initialized a Gaussian Process with a Matérn kernel (*ν* = 1.5) and initial length scales of [1, 20, 10] for amplitude, frequency, and pulse width, respectively. These settings reflect our initial assumptions regarding the function’s smoothness and the rate at which correlation diminishes as input points diverge. All hyperparameters were subsequently optimized based on the observed data by maximizing the log-marginal likelihood in *scikit-learn*’s “GaussianProcessRegressor”, ensuring that the final kernel parameters accurately captured the underlying relationships in the training set. Specifically, we set the number of restart optimizers to 40 to reduce the risk of convergence to a suboptimal local minimum. This procedure yielded the final optimized length scales for subjects 1, 2, and 3 as [0.85, 9.91, 1.44], [1.1, 30.24, 10.12], and [5.03, 98.45, 202.1], respectively. Ultimately, we validate the model by evaluating these optimized settings and comparing them to their clinical configurations. We implement this approach using the *scikit-learn* library^112^, which enables us to develop a nonlinear model that predicts the WPI based on DBS settings and quantifies the uncertainty of these predictions. The approach is particularly well-suited for our objective of generating personalized maps of DBS settings to WPI, offering insights into the optimal stimulation parameters for enhancing walking performance.

To evaluate the relationship between WPI and feedback rankings (both from patients and physical therapists), we employed a linear mixed-effects model to capture the overall association across subjects while including a random intercept for each participant to handle within-subject clustering. In this model, the ranked WPI values served as the fixed effect, and the feedback rankings were treated as the outcome variable. This approach preserved the advantages of analyzing ordinal-like data while properly addressing the dependence among repeated observations for each participant, thereby yielding a more reliable estimate of the relationship between WPI and subjective feedback. Within each visit, WPI values were ranked such that the highest WPI value received a rank of 1, with the ranking continuing in descending order. This provided a set of ranked WPI values for each visit. The ranked WPI values and the corresponding feedback rankings from both patients and physical therapists across all subjects were combined into a single dataset for comprehensive analysis.

### Neural data processing and neurophysiological analysis

To identify the neurophysiological basis for changes in these gait metrics, we analyzed pallidal LFP fluctuations, cortical activity, and pallidal-cortical coherence dynamics (i.e., wavelet coherence) during each gait cycle as patients walked back and forth during the walking performance evaluation. To synchronize the neural data with the gait kinematics recorded by the IMU sensors, we utilized peak acceleration moments as temporal anchors. Specifically, peak accelerations were identified from both the IMU data attached to the participant’s chest and the measurements from the Summit RC+S built-in accelerometer. Once identified, these peak acceleration moments were used to align the neural signal data from the Summit RC+S device with the corresponding gait kinematics. The detailed synchronization process is illustrated in Supplementary Fig. 7. To assess the consistency of peak acceleration timing and amplitude across different DBS parameters and participants, we performed an analysis of the distribution of peak acceleration amplitudes and the intervals between consecutive peaks. This analysis is illustrated in Supplementary Fig. 8, which displays the distribution of peak acceleration amplitudes and inter-arrival times. The findings indicate a consistent pattern of peak accelerations across participants, reinforcing the reliability of our synchronization method despite the small sample size.

Neural data were sampled at 500 Hz and processed through a 1 Hz high-pass filter. Neural data streamed from the Summit RC+S were time-synchronized to wearable devices that captured heel-strike and toe-off events. We then performed time-frequency and time-varying coherence analyses on each pallidal contact pair and the two cortical channels during all gait cycles, excluding turns, as standard gait cycle events do not occur during turning. We used MATLAB’s built-in continuous wavelet transform function *(cwt)* for time-frequency analysis. Additionally, we conduct a comprehensive analysis to examine the interaction between cortical and subcortical areas within each hemisphere. This includes coherence between the LFP recordings from the pallidum and ECoG recordings from the motor cortex within each brain hemisphere. Because gait involves continuously changing neural dynamics, we employed wavelet coherence to track time-varying coupling between cortical activity and basal ganglia during walking. Unlike magnitude-squared metrics designed for stationary signals, wavelet coherence can resolve transient synchrony across frequencies. Specifically, we used MATLAB’s built-in *(wcoherence)* function to compute high-resolution, time-frequency coherence estimates between GP LFP and the two motor-cortical channels. To ensure the accuracy of our analysis, we meticulously removed artifacts, including motion, electrocardiogram-related, and stimulation artifacts. We addressed potential EKG-induced artifacts by implementing an adaptive EKG artifact removal technique, ensuring accurate identification of EKG artifact instances^113^. Significant EKG artifacts were not prevalent in the conventional sensing channels within this cohort. However, when EKG-like artifacts were detected in stimulation contacts, we carefully analyzed these instances and cross-referenced them with other channels to confirm the absence of EKG artifacts in the LFP recordings. We also employed a multi-step approach to detect unusual high-frequency (gamma-band) activity. First, we applied a Butterworth high-pass filter at 4 Hz (order = 4) in the *FieldTrip* toolbox to eliminate low-frequency drift; this step was used solely to identify outliers in the 75–150 Hz gamma range. We then computed *z-scores* and flagged any segments exceeding an 8 *z-score* threshold as potential artifacts, placing a 200 ms blanking buffer around each flagged segment and replacing these intervals with NaNs. Subsequently, for our primary analyses focusing on the 1–30 Hz canonical frequency bands, we retained frequencies down to 1 Hz. As a result, all canonical frequency bands within 1–30 Hz remained intact for our subsequent neural analyses. Additionally, we mitigated the possibility of data packet loss in the Summit RC+S devices by identifying such events through low-frequency analysis, thereby excluding the affected gait events from further analyses.

Following artifact removal, we analyzed the spectral power within each frequency band and normalized the results across all tested DBS settings during each patient visit. We used the *z-score* method for each frequency to normalize the signal power and coherence values within each visit. Due to the presence of stimulation-induced sub-harmonic activity in some DBS settings, our analysis focused on the 1–30 Hz canonical frequency bands Specifically, we defined non-overlapping bands as follows: delta (2–4 Hz), theta (4–8 Hz), alpha (8–12 Hz), and beta (12–30 Hz), where the lower bound is inclusive and the upper bound is exclusive for each band. Supplementary Fig. 9 illustrates an example of power spectral density and *cwt* of the LFP signal in the GP region under 60 Hz and 145 Hz stimulation conditions for Subject 3. By restricting our analysis to the 1–30 Hz frequency range, we ensured that stimulation-induced artifacts and their subharmonics were excluded from subsequent analyses (Supplementary Fig. 9). Next, we extracted single gait trials from the clean, normalized time-frequency representations of neural recordings, and sorted the gait cycles by their stride times. Within these frequency bands, we compute the average levels of signal power during phases of gait cycles across all trials (i.e., exemplified in Fig. 4). Next, to assess the impact of variations in DBS settings on the neural signal power across different gait phases, we determined the average levels of signal power for each canonical frequency band during each gait phase. Different phases include two double limb support periods and two swing phases (i.e., RTO to RHS for right leg swing and LTO to LHS as left leg swing).

### Correlation between neural oscillations and improved walking performance

To determine the neurophysiological basis underlying changes in gait metrics, we analyzed fluctuations in pallidal LFPs, cortical activity, and pallidal-cortical coherence dynamics throughout each gait cycle as participants walked back and forth during the walking performance assessment. We then used linear mixed-effects models to evaluate neural biomarkers of walking performance. This approach included fixed effects for neural features, which represented the influence of neural oscillations on walking performance, and random effects for each individual to account for inter-subject variability. Given the exploratory nature of our analysis, we assessed correlations between individual neural features and walking performance independently, rather than constructing a comprehensive model incorporating all potential features simultaneously. Our aim was to identify consistent neural biomarkers across all participants that correlated with walking performance.

Neural features analyzed included the average levels of spectral power in the canonical frequency bands and coherence between cortical and subcortical regions. These analyses were performed during different phases of the gait cycle. Additionally, we conducted both group-level and person-specific analyses to identify shared neural markers across the cohort and individual-specific analyses to capture unique characteristics that contributed to improved walking performance under gait-optimized DBS settings. This dual approach ensured a comprehensive understanding of the common and individual neural dynamics linked to gait enhancement.

For person-specific analysis, data from each subject were aggregated across all tested DBS settings. For the spectral power analysis, separate ordinary linear regression models were fit for each electrode contact (i.e., one subcortical and two cortical recordings) and for each canonical frequency band below 30 Hz. In these models, the average power levels across the four gait phases were used as predictors of the WPI, with the hemisphere included as a fixed effect. To control for multiple comparisons within each model, *p*-values were adjusted using the BH method. A similar approach was employed for the coherence analysis: regression models were fit for each electrode pair (two subcortical-cortical pairs and one inter-cortical pair) and each canonical frequency band, using coherence measurements across gait phases as predictors, with BH adjustment applied to the corresponding *p*-values. This methodological framework enabled us to extract robust, person-specific neural biomarkers that reflect the unique oscillatory profiles associated with improved walking performance.

For group-level analysis, to identify shared neural biomarkers associated with walking performance across individuals, we employed a linear mixed-effects model. For each spectral feature (defined as the average power within a canonical frequency band and across each specific gait phase), a separate model was fit that included the feature of interest and hemisphere as fixed effects, along with a random intercept for each subject to account for inter-individual variability. This design also tests whether a feature’s link to WPI depends on the hemisphere while still accounting for variability between subjects. In these models, the average spectral power was extracted from all canonical bands and across all gait cycle epochs and correlated with the WPI measured over multiple visits and DBS settings. We evaluated the contribution of each feature by comparing its full model to a corresponding null model (excluding the feature) via *ANOVA*. In addition, we utilized the stepwise model selection procedure available in the *lme4* package (via the *step* function) to fit a comprehensive model that initially included all features. By sequentially removing each feature and assessing the change in model fit, we identified those features that significantly improved the model. The significant features derived from both the *ANOVA*-based comparisons and the stepwise selection approach were consistent, thereby validating our findings. To control for multiple comparisons across channels, *p*-values were adjusted using the BH method. This systematic approach enabled us to isolate robust neural signatures that predict gait performance at the group level, while simultaneously accounting for clinical heterogeneity and variability in DBS parameters.

### Validation of cortical electrode coverage for gait activity

To evaluate whether the limited coverage of the cortical electrode strip could capture leg-related motor activity during walking, we incorporated an additional experimental task. During research visits, participants were instructed to perform arm swing movements while standing, without walking, for ~1 min. These isolated arm swing cycles were synchronized with the gait cycles recorded during overground walking trials. Neural activity and gait kinematics were concurrently recorded using the same electrode setup. The synchronization ensured that comparable motor activities could be analyzed under controlled conditions. Wavelet transform analysis was subsequently applied to both walking and isolated arm swing data to examine distinct patterns of signal power associated with each motor activity. Our wavelet transform analysis revealed that cortical signal power during overground walking significantly differs from that during isolated arm swings while standing (Supplementary Figs. 10–12). These results demonstrate that our cortical electrode strip effectively records coordinated motor activity involving both legs and arms during walking.

The analysis pipeline incorporated multiple software environments. MATLAB was used for preprocessing, signal filtering, neural data alignment, and feature extraction; R (using the *lme4* package) was employed for statistical modeling and linear mixed-effects regression; and Python (with the *scikit-learn* library) was used for machine learning-based modeling and parameter optimization.

## Supplementary information

Supplementary Information

consort checklist

## Data Availability

Data from this study can be made available upon reasonable request, provided that patient confidentiality is maintained and disclosure standards are met.
