# Supplementary material for: Modeling and optimizing deep brain stimulation to enhance gait in Parkinson’s disease: personalized treatment with neurophysiological insights
Source: NPJ Parkinsons Dis. 2025 Jun 18;11:173. doi: 10.1038/s41531-025-00990-5 (PMC12177069; doi:10.1038/s41531-025-00990-5)
Supplement: Supplementary file 1 — Supplementary Information [file 41531_2025_990_MOESM1_ESM.pdf]

Modeling and Optimizing Deep Brain Stimulation to Enhance Gait in Parkinson's Disease: Personalized Treatment with Neurophysiological Insights

Hamid Fekri Azgomi[1], Kenneth H. Louie[1], Jessica E. Bath[1,2],
Kara N. Presbrey[1], Jannine P. Balakid[1], Jacob H. Marks[1],
Thomas A. Wozny[1], Nicholas B. Galifianakis[3],
Marta San Luciano[3], Simon Little[3], Philip A. Starr[1],
Doris D. Wang[1]

[1]Department of Neurological Surgery.
[2]Department of Physical Therapy and Rehabilitation Science.
[3]Department of Neurology,
University of California San Francisco, California, USA.

Supplementary materials

Supplementary Figures 1. - 12. are presented as supplementary information.

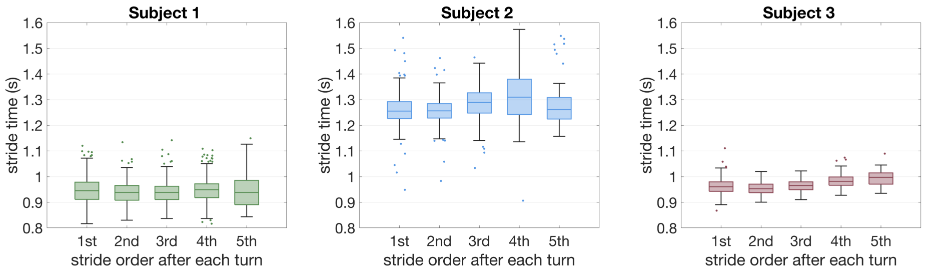

Supplementary Figure 1. Stride time consistency across stride orders during a 6-meter walk

Each panel represents an individual participant. Boxplots display the distribution of stride times categorized by stride order during a 6-meter overground walk. The x-axis denotes the different stride orders, while the y-axis indicates stride time in seconds.

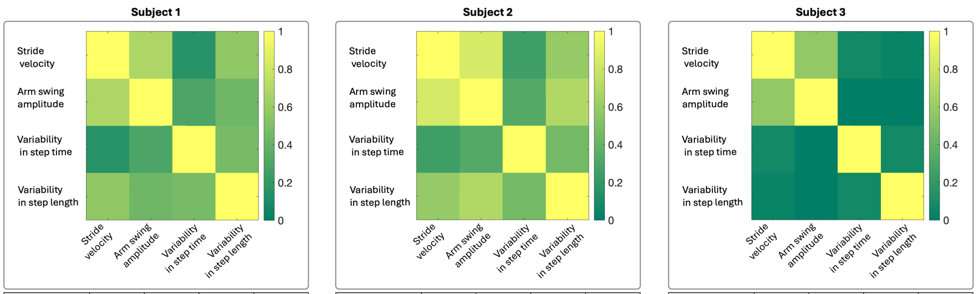

metric	Stride velocity	Arm swing amplitude	Variability in step time	Variability in step length
Variance Inflation Factor (VIF)	2.42	1.99	1.41	1.90
The average Δ in WPI (in response to a 10% decrease in metric)	-2.72	-3.25	-2.21	-1.82
The average Δ in WPI (in response to a 10% increase in metric)	2.71	3.24	0.82	0.61

metric	Stride velocity	Arm swing amplitude	Variability in step time	Variability in step length
Variance Inflation Factor (VIF)	3.31	4.17	1.26	2.17
The average Δ in WPI (in response to a 10% decrease in metric)	-2.81	-3.39	-1.93	-1.86
The average Δ in WPI (in response to a 10% increase in metric)	2.78	3.37	0.73	0.76

metric	Stride velocity	Arm swing amplitude	Variability in step time	Variability in step length
Variance Inflation Factor (VIF)	1.51	1.49	1.02	1.01
The average Δ in WPI (in response to a 10% decrease in metric)	-2.85	-3.13	-1.92	-2.01
The average Δ in WPI (in response to a 10% increase in metric)	2.84	3.12	0.71	0.72

Supplementary Figure 2. Correlation, variance, and sensitivity analysis of gait metrics with WPI for each participant

Each panel displays the pairwise Pearson correlation coefficients among all gait metrics and the WPI for an individual participant. The matrix visualizes the degree of linear relationships between variables, with color intensity representing the strength of the correlations: colors range from green (lower values) to yellow (higher values). The low off-diagonal values (green shades) indicate minimal multicollinearity among the metrics, confirming that each metric provides unique and independent information contributing to the WPI. Additionally, each panel includes a table presenting the results of variance inflation factor (VIF) analysis and a sensitivity investigation. The sensitivity analysis was performed by applying a ±10% change to each metric to assess their impact on the WPI.

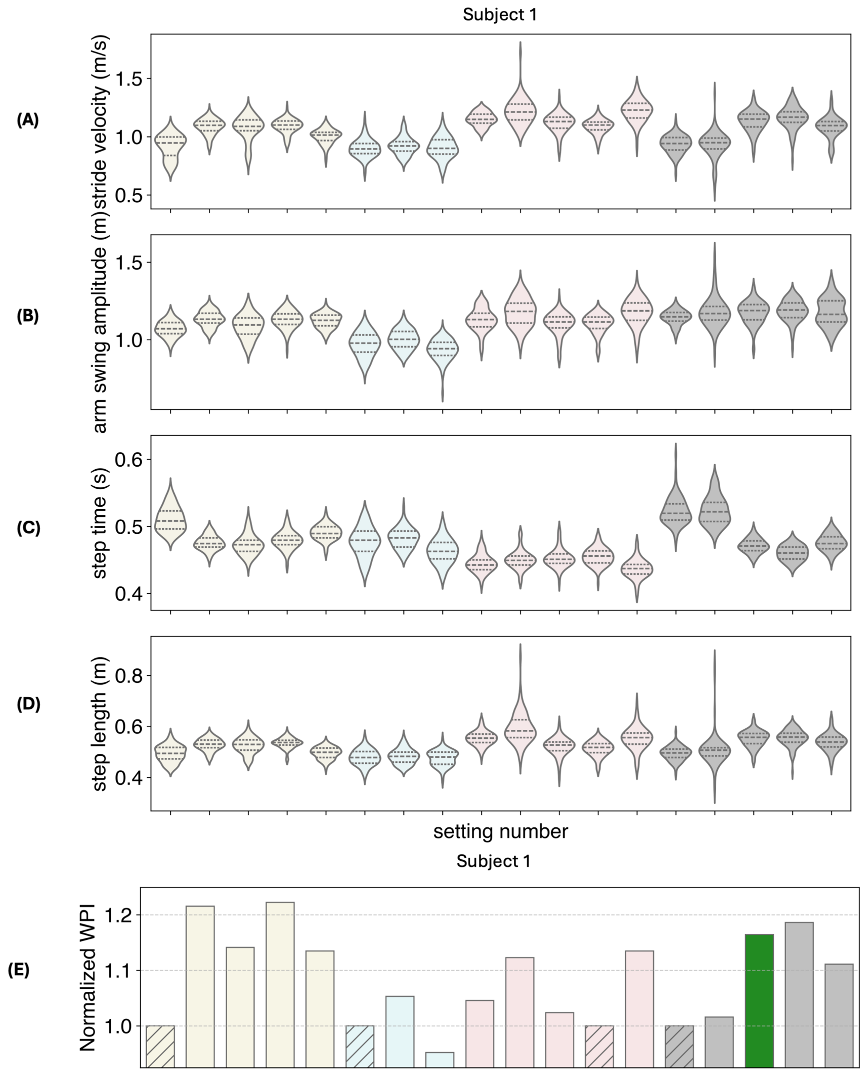

Visit	Visit 1					Visit 2			Visit 3					Visit 4 (test & validation)				
Setting	1	2	3	4	5	6	7	8	9	10	11	12	13	14	15	16	17	18
Frequency	150	150	60	60	190	150	190	190	150	60	60	150	190	150	190	60	125	190
Amplitude	5.5	4.1	4.1	5.5	5.5	5.5	5.5	4.1	4.1	4.1	5.5	5.5	4.1	5.5	5.5	5.1	4.1	5.5
Pulse width	90	90	90	90	90	90	70	70	90	90	90	90	70	90	70	90	80	80

Supplementary Figure 3. Impact of DBS setting adjustments on gait kinematics and walking performance (subject 1).

Panels **(A)**-**(D)** illustrate the variations in gait kinematics as a response to different DBS settings across three visits, measured by **(A)** stride velocity, **(B)** arm swing amplitude, **(C)** step length variability, and **(D)** step time variability. Panel **(E)** displays the corresponding changes in the walking performance index (WPI). Columns with hatched patterns represent the clinically optimized DBS settings tested at each visit. In Visit 4, the green-colored bars correspond to the DBS settings predicted by the Gaussian Process Regression (GPR) model to enhance walking performance. The detailed stimulation parameters for each setting are summarized in the table at the bottom, with each color representing data from a specific visit.

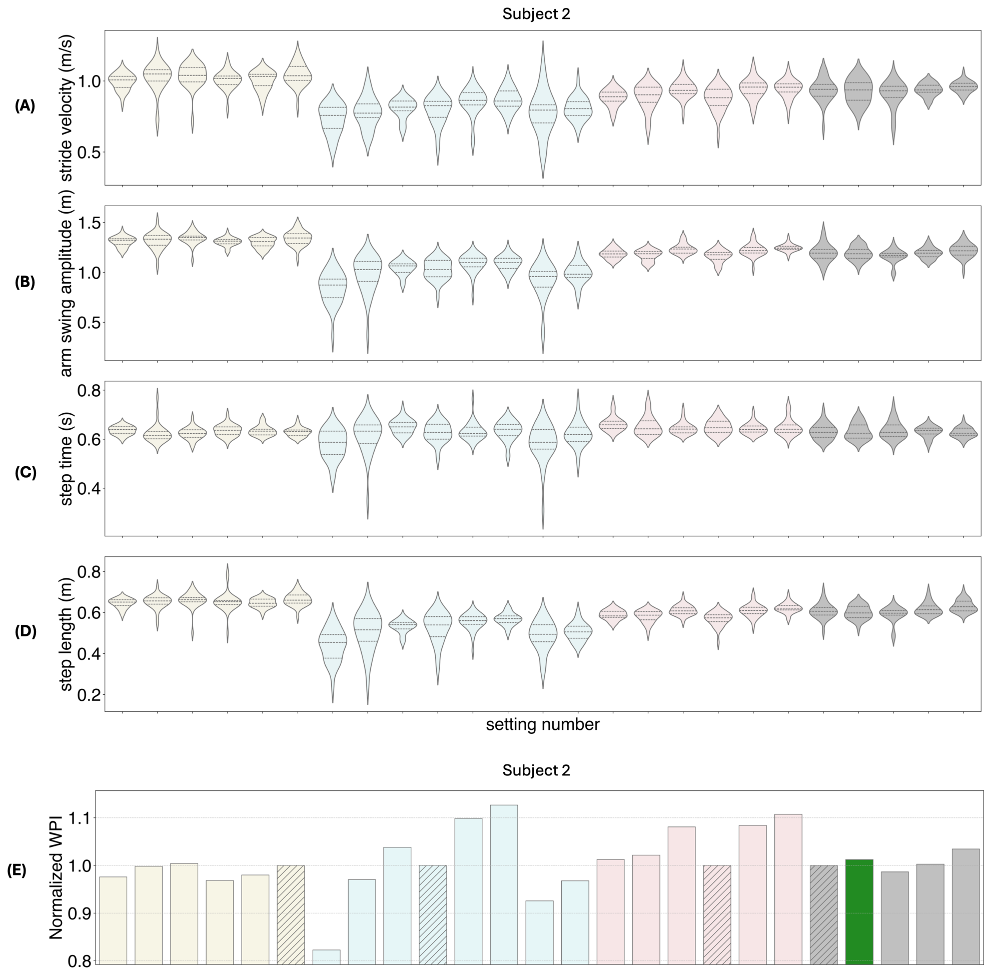

Visit	Visit 1						Visit 2								Visit 3						Visit 4 (test & validation)				
Setting	1	2	3	4	5	6	7	8	9	10	11	12	13	14	15	16	17	18	19	20	21	22	23	24	25
Frequency	130	60	60	180	180	130	130	60	60	130	60	60	130	130	130	60	60	130	60	180	130	60	60	180	180
Amplitude	2.8	2.8	4.0	4.0	2.8	4.0	4.0	4.0	4.0	4.0	5.0	5.0	5.0	5.0	4.0	4.0	2.8	4.0	2.8	4.0	4.0	4.0	4.8	4.0	2.8
Pulse width	60	60	60	60	60	60	70	70	60	60	60	70	70	60	70	70	70	60	60	60	60	60	70	60	60

Supplementary Figure 4. **Impact of DBS setting adjustments on gait kinematics and walking performance (subject 2)**.

Panels **(A)**-**(D)** illustrate the variations in gait kinematics as a response to different DBS settings across three visits, measured by **(A)** stride velocity, **(B)** arm swing amplitude, **(C)** step length variability, and **(D)** step time variability. Panel **(E)** displays the corresponding changes in the walking performance index (WPI). Columns with hatched patterns represent the clinically optimized DBS settings tested at each visit. In Visit 4, the green-colored bars correspond to the DBS settings predicted by the Gaussian Process Regression (GPR) model to enhance walking performance. The detailed stimulation parameters for each setting are summarized in the table at the bottom, with each color representing data from a specific visit.

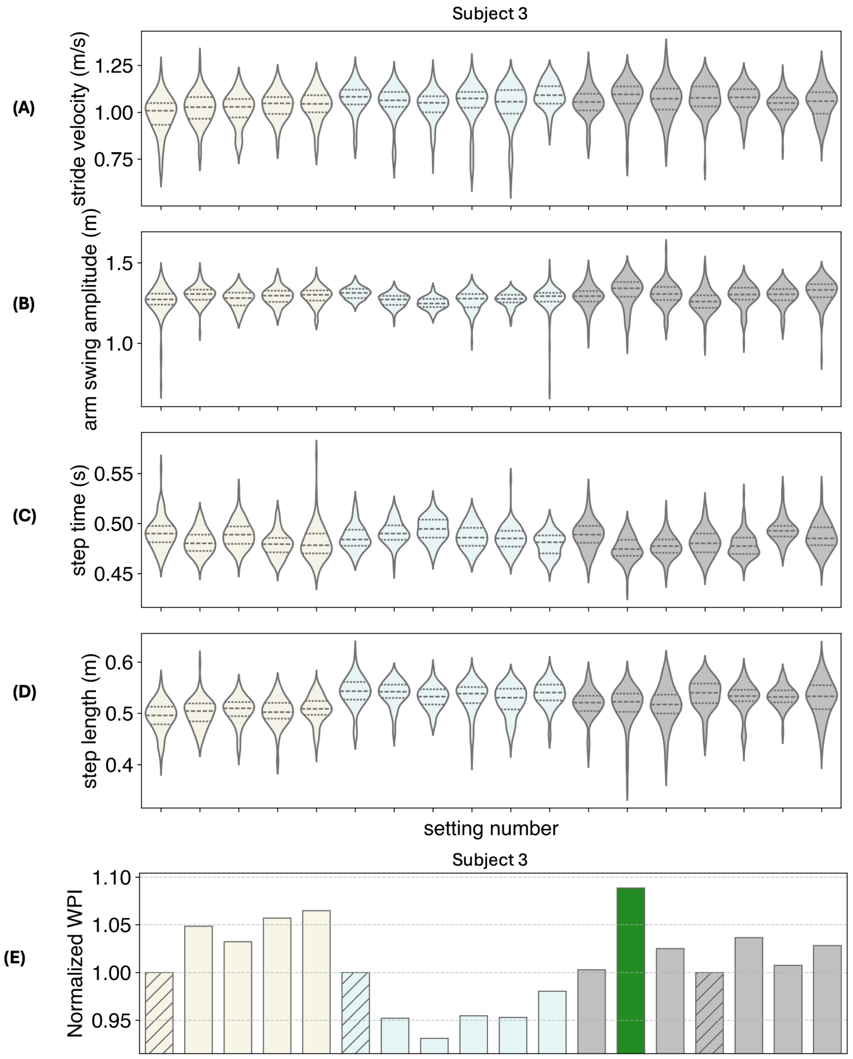

Visit	Visit 1					Visit 2						Visit 3 (test & validation)						
Setting	1	2	3	4	5	6	7	8	9	10	11	12	13	14	15	16	17	18
Frequency	130	60	60	130	180	130	60	60	130	180	180	60	180	145	145	190	60	145
Amplitude	4.7	4.7	3.5	3.5	3.5	4.9	4.9	3.6	3.6	3.6	4.9	3.9	4.2	3.0	3.9	3.5	3.9	4.9
Pulse width	60	60	60	60	60	60	60	80	80	80	60	60	80	60	60	80	80	80

Supplementary Figure 5. Impact of DBS setting adjustments on gait kinematics and walking performance (subject 3).

Panels **(A)**-**(D)** illustrate the variations in gait kinematics as a response to different DBS settings across three visits, measured by **(A)** stride velocity, **(B)** arm swing amplitude, **(C)** step length variability, and **(D)** step time variability. Panel **(E)** displays the corresponding changes in the walking performance index (WPI). Columns with hatched patterns represent the clinically optimized DBS settings tested at each visit. In Visit 3, the green-colored bars correspond to the DBS settings predicted by the Gaussian Process Regression (GPR) model to enhance walking performance. The detailed stimulation parameters for each setting are summarized in the table at the bottom, with each color representing data from a specific visit.

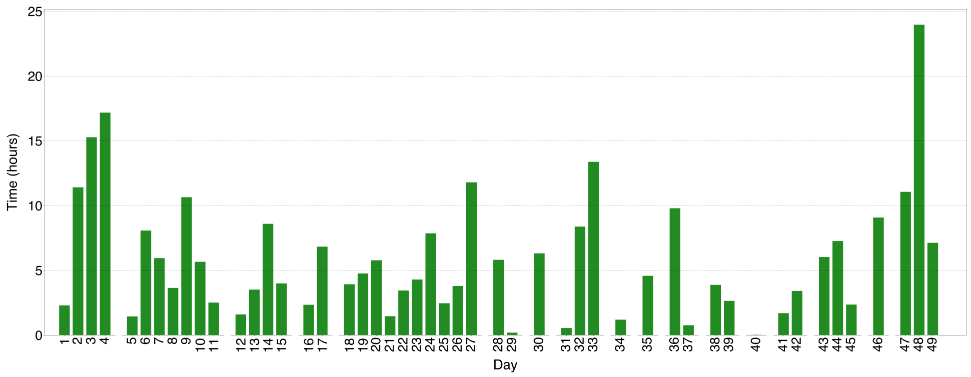

Supplementary Figure 6. Time spent on gait-optimized setting (subject 1).

The bar chart illustrates the total time intervals measured across consecutive days. Sequential day numbers are displayed on the x-axis for days with recorded data. Gaps in the data are visually represented by open spaces on the x-axis, indicating periods where no changes to the DBS settings were made. The y-axis represents the total time interval (in hours) for each day.

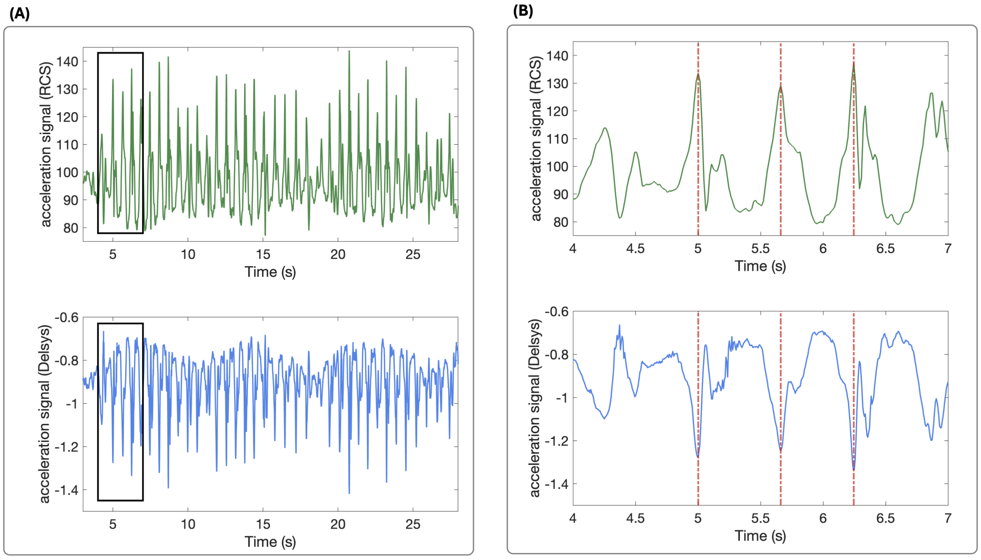

Supplementary Figure 7. Synchronization of neural and gait kinematics using peak acceleration moments.

Panel **(A)** displays a zoomed-out view of acceleration signals recorded during a 6-meter overground walk. The top trace represents data from the RCS device, while the bottom trace shows data from an external chest-mounted accelerometer positioned adjacent to the pulse generator. Panel **(B)** presents a zoomed-in view of the same acceleration signals, highlighting the peak moments used as temporal anchors for synchronizing neural data with gait kinematics. The alignment of peak accelerations from both devices ensures accurate correspondence between neural activity and gait measurements, validating the reliability of our synchronization method.

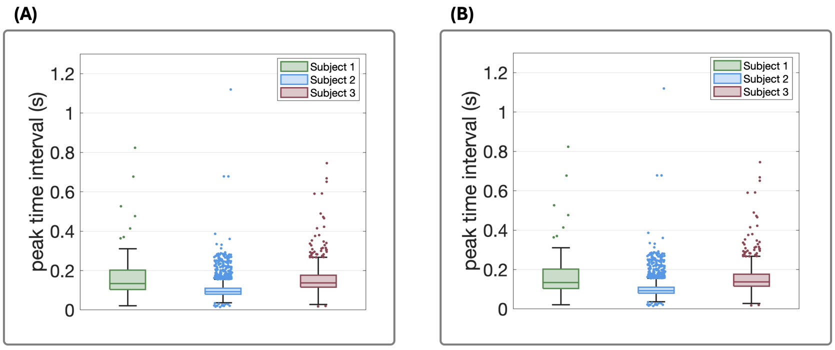

Supplementary Figure 8. Distribution of peak acceleration amplitudes and inter-arrival times across DBS settings.

Panel **(A)** presents boxplots illustrating the distribution of peak acceleration amplitudes across all DBS settings for each participant. Panel **(B)** displays boxplots of the inter-arrival times between consecutive peak acceleration moments for each participant. Each panel includes three boxplots corresponding to individual participants, highlighting the consistency of peak acceleration patterns across different DBS parameters. The uniform distributions observed in both panels confirm that peak acceleration timing and amplitude remain stable across DBS settings, reinforcing the reliability of our synchronization method.

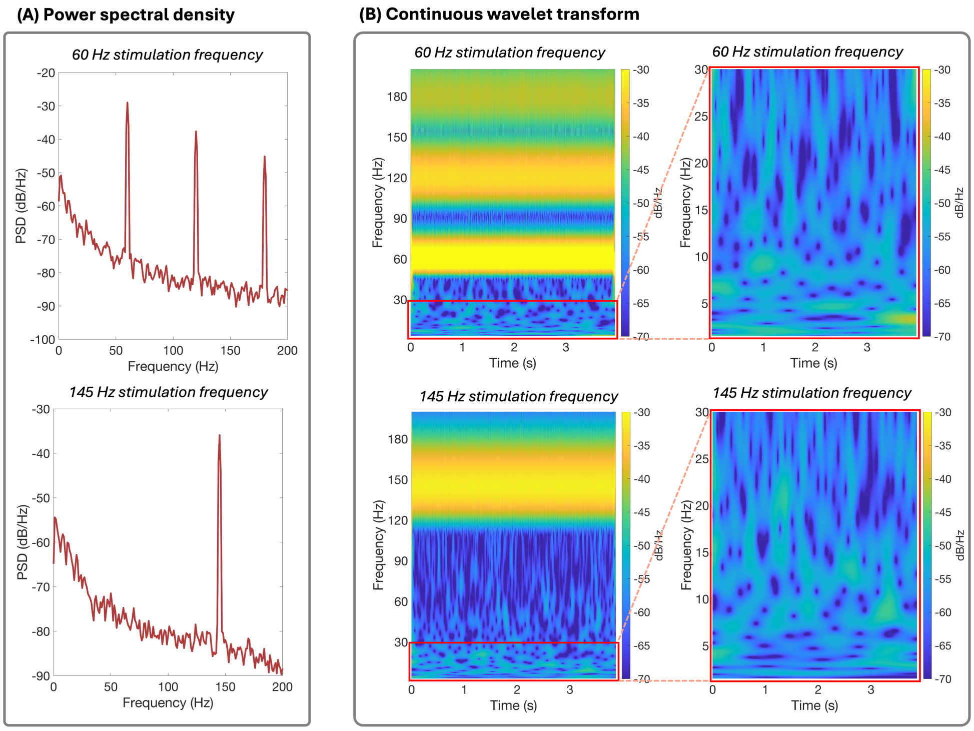

Supplementary Figure 9. Impacts of stimulation-induced artifacts in time-frequency analysis.

Panel **(A)** illustrates the power spectral density of the LFP signal from the GP under two stimulation conditions. The top sub-panel shows the results at a stimulation frequency of 60 Hz, while the bottom sub-panel shows results at 145 Hz. Panel **(B)** presents the continuous wavelet transformation of the GP signal, with the top and bottom sub-panels corresponding to the 60 Hz and 145 Hz stimulation frequencies, respectively. The right sub-panels in panel **(B)** display a zoomed-in view of the frequency range between 0 and 30 Hz, highlighting the focus of the analysis on avoiding stimulation-induced artifacts in this lower frequency band.

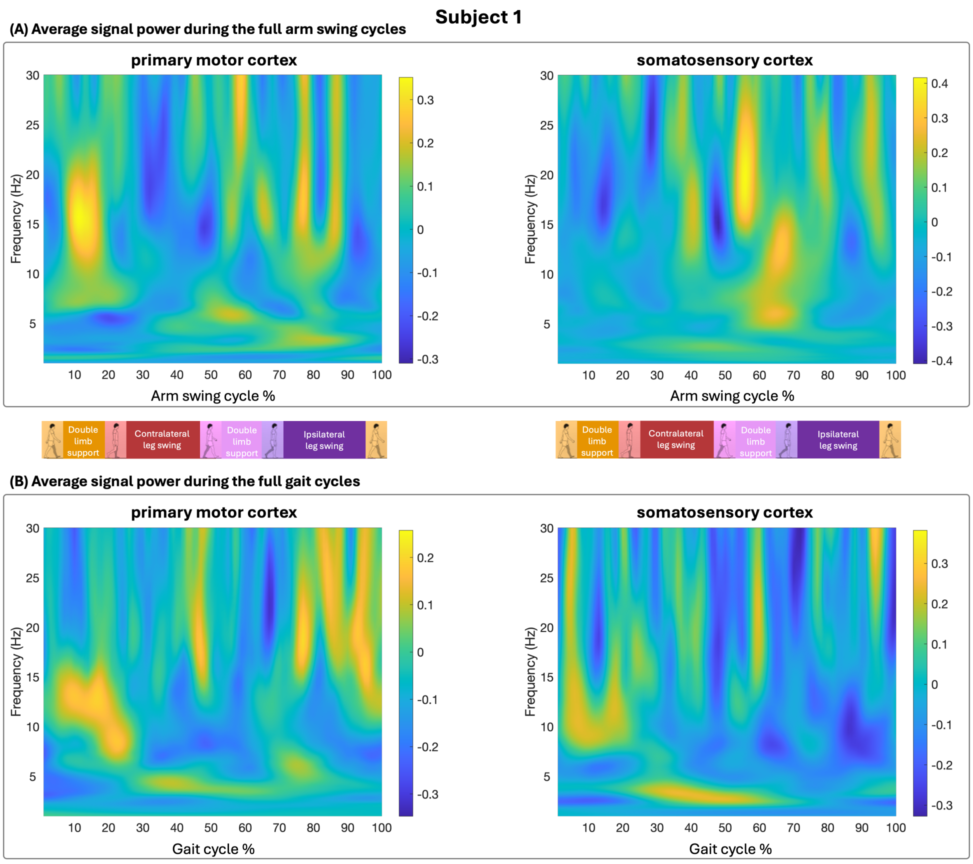

Supplementary Figure 10. Cortical signal activity during standing arm swings and overground walking (Subject 1).

Panel **(A)** displays the average continuous wavelet transform of cortical signals recorded during full arm swing cycles. Panel **(B)** shows the average continuous wavelet transform of cortical signals recorded across all gait cycles during overground walking. Within each panel, the left and right sub-panels represent data from the primary motor cortex and the somatosensory cortex, respectively. The distinct patterns observed indicate that the cortical electrode strip effectively captures motor activity related to both arm and leg movements during walking.

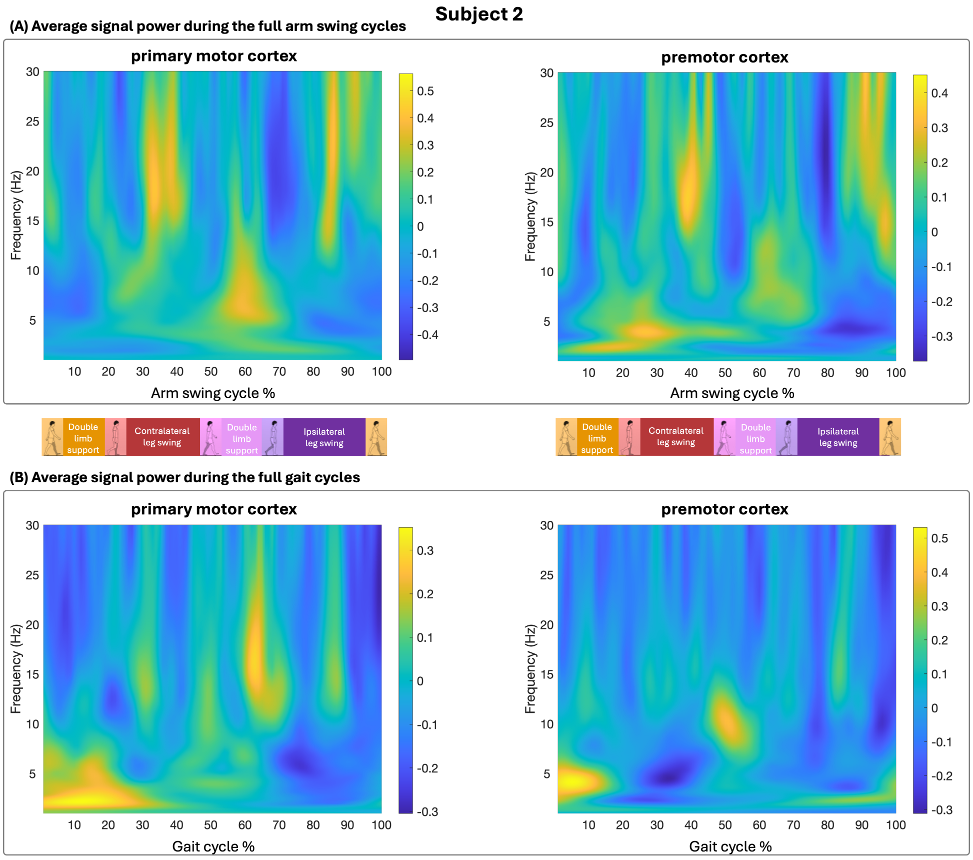

Supplementary Figure 11. Cortical signal activity during standing arm swings and overground walking (Subject 2).

Panel **(A)** displays the average continuous wavelet transform of cortical signals recorded during full arm swing cycles. Panel **(B)** shows the average continuous wavelet transform of cortical signals recorded across all gait cycles during overground walking. Within each panel, the left and right sub-panels represent data from the primary motor cortex and the somatosensory cortex, respectively. The distinct patterns observed indicate that the cortical electrode strip effectively captures motor activity related to both arm and leg movements during walking.

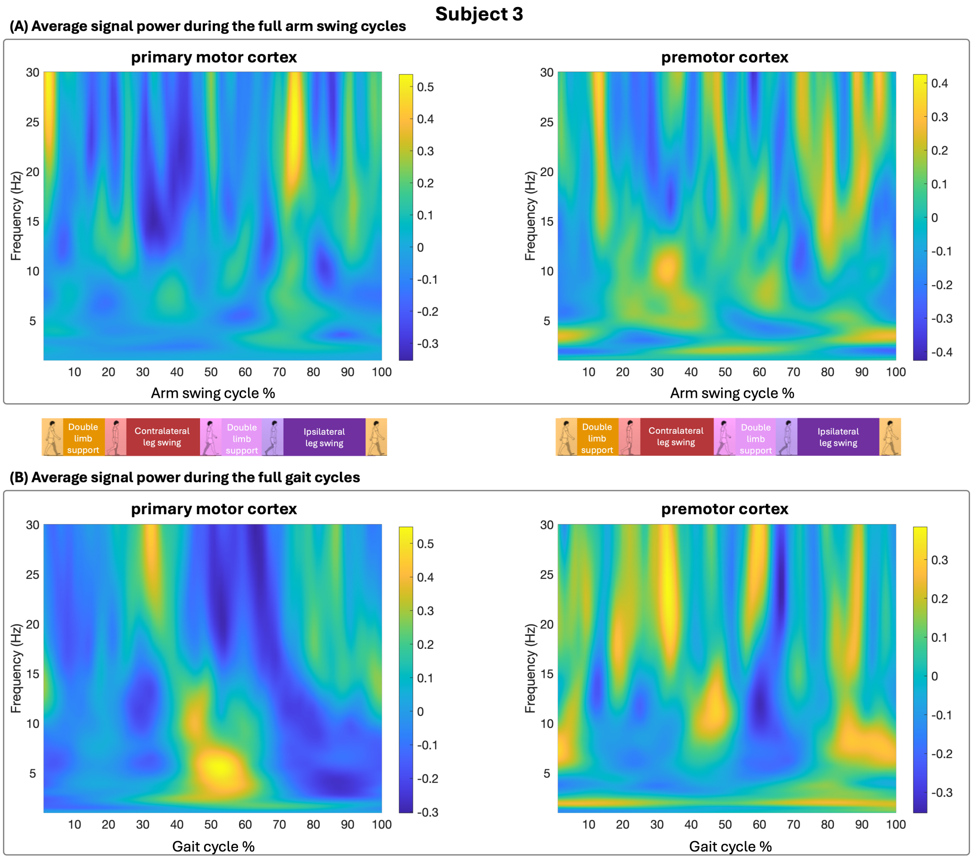

Supplementary Figure 12. Cortical signal activity during standing arm swings and overground walking (Subject 3).

Panel **(A)** displays the average continuous wavelet transform of cortical signals recorded during full arm swing cycles. Panel **(B)** shows the average continuous wavelet transform of cortical signals recorded across all gait cycles during overground walking. Within each panel, the left and right sub-panels represent data from the primary motor cortex and the somatosensory cortex, respectively. The distinct patterns observed indicate that the cortical electrode strip effectively captures motor activity related to both arm and leg movements during walking.
